# Sexual Dimorphism in Allergic and Mast Cell-Associated Diseases

**DOI:** 10.1007/s12016-026-09150-w

**Published:** 2026-04-11

**Authors:** Aditya Kotha, Tania D. Maldonado, Hadi Hamze, Zakaria Y. Hussain, Navya Gobinathan, An Z. Yao, Mahamed Abudulkadir, John M. Ching, John J. Ryan

**Affiliations:** School of Life Sciences and Sustainability, Virginia Commonwealth University, Richmond, VA 23284 USA

**Keywords:** Allergy, Sexual dimorphism, Inflammation, Allergic inflammation, IgE

## Abstract

Allergic diseases are common, affecting more than one-third of the global population and varying widely in severity. A consistent theme in asthma, food allergy, atopic dermatitis, and chronic urticaria is a female-dominant prevalence in adulthood. Some of these begin with male predominance in childhood and shift with puberty. A smaller subset of allergic diseases, including eosinophilic esophagitis and vernal keratoconjunctivitis, has a clear male prevalence. In fact, nearly all allergic diseases exhibit sexual dimorphism in either prevalence or severity. These striking disparities are being unraveled to reveal mechanisms that might be clinically targeted. In this review, we discuss the current state of knowledge about sexual dimorphism for these important inflammatory disorders. These insights are foundational for optimizing personalized medicine.

## Introduction

Sexual dimorphism is defined as differences in biological characteristics between male and female members of the same species. The presence of sexual dimorphism results from a complex relationship between evolution and genetics [1]. Genetics determines the formation of gonads within an organism. The timing with which gonadal androgens and estrogens reach their peak is also sexually dimorphic. Males experience a surge in testosterone early in life, while estrogen increases later in females [2].

Sex hormones have a significant impact on the immune system. Estrogen receptors (ERs) are expressed by many immune cells, including lymphocytes, macrophages, eosinophils, basophils, dendritic cells, mast cells, and B and T cells [3]. In high concentrations, estrogen encourages a Th2 response and aids B cell class switching to IgE [4, 5]. It can also promote mast cell and basophil degranulation [4]. Androgen receptors (ARs) are expressed in hematopoietic stem cells, lymphoid and myeloid progenitor cells, and cells from the bone marrow, thymus, and spleen [5, 6]. Unlike ERs, AR signaling typically suppresses the immune response [3], inhibiting both Th1 and Th2 responses [7]. Testosterone also reduces B cell number [8]. These effects make males generally less susceptible to inflammatory and autoimmune diseases [3].

In addition to sex-specific hormones, chromosomal expression differs between males and females. The female XX chromosomal pair undergoes incomplete X chromosome inactivation. However, ~ 15% of the 1100 genes encoded in the human X chromosome evade inactivation and can contribute to sexual dimorphism by yielding higher gene expression in females [9, 10]. For example, the innate inflammatory receptors TLR7 [11] and TLR8 [12] have been shown to escape X-inactivation. Likewise, the male-restricted Y-chromosome genes *KDM5D* [13] and *UTY* [14] have been linked to reduced inflammation, while the male gonad-determining gene *SRY* promotes [15] inflammation.

Interestingly, X chromosome inactivation itself promotes sexually dimorphic inflammation. The long non-coding RNA *Xist* is transcribed from the X chromosome only in females and generates a ribonucleoprotein complex used to inactivate the chromosome. This ribonucleoprotein includes antigenic components that can induce autoantibodies in mice. Transgenic expression of RNA *Xist* in males is sufficient to generate autoantibodies and reproduce female autoimmunity [16].

There is evidence of sexual dimorphism in type 2 (T2) inflammatory and allergic diseases [17]. An overactive T2 response can induce allergic diseases through the actions of epithelial cells, dendritic cells, T cells, innate lymphoid cells, eosinophils, mast cells, and basophils. Mechanisms include variations in the Th2 cytokines, interleukin (IL)−4, IL-5, IL-9, IL-13, and IL-31 [18]. Females may be predisposed to T2 inflammation due to estrogen effects. For example, a recent study showed that estrogen increases memory B cell production selectively in XX-karyotype individuals [19].

T2 allergic diseases include asthma, chronic rhinosinusitis with nasal polyps (CRSwNP), allergic rhinitis, atopic dermatitis, chronic pruritus, eosinophilic esophagitis, food allergy, allergic conjunctivitis, chronic urticaria, and mast cell activation syndrome [20–26]. These inflammatory disorders can emerge in childhood or adulthood and greatly affect quality of life [27]. It is important to understand disease pathophysiology, which can be partly due to sexual dimorphism. Our aim in this review is to summarize the available literature describing sexual dimorphism in allergic disease.

### Atopic Dermatitis

Atopic dermatitis (AD) is a common condition characterized by chronic pruritus and inflammation of the skin, with a range of severity [28]. AD has become an increasingly relevant health concern, affecting > 200 M people globally, or approximately 2.5% of the population [29, 30].

AD development is related to environmental, genetic, and immunological triggers [31, 32]. Interestingly, AD is part of the “atopic march”, associated with asthma and chronic rhinitis. In this model, AD has the highest incidence rates in infancy, followed by a rise in asthma in adolescence, and allergic rhinitis after puberty [33]. AD severity varies widely, which can prove challenging for treatment. While a unified grading scale has not been established, severity indices can be used to quantify disease intensity, including the Eczema Area and Severity Index (EASI) and the SCORing Atopic Dermatitis (SCORAD) index [34, 35]. Recent treatment breakthroughs have made AD management and long-term remission more attainable.

#### AD Pathophysiology

Due to the prominent physical symptoms of the disease, AD remains a largely descriptive diagnosis. Acute (< 72-hour-old) lesions typically appear bright red and have exudate with little skin thickening. Chronic lesions are typically dull red, dry/scaly, and have thickened skin. Biomarkers such as elevated IgE levels, eosinophilia, and increased type 2 Th cell (Th2) cytokines including IL-4, IL-5, IL-13, and IL-31 are associated with AD pathogenesis [36–38]. A subset of AD patients (∼20%) shows normal IgE levels and no evidence of allergy. This is sometimes referred to as “intrinsic” AD [39, 40]. Progression from acute to chronic lesions has been ascribed to a transition from a largely Th2 response to a mixed Th1/Th17/Th22 response [41]. However, two RNA sequencing-based studies comparing non-lesional skin to acute and chronic AD lesions concluded that acute versus chronic lesions are largely distinguished by two features: a quantitatively greater inflammatory cytokine response and a qualitative shift to cellular repair and regeneration gene induction in late lesions [42, 43]. A unified view is that the acute-to-chronic transition is marked by enhanced cytokine responses that promote skin thickening through factors such as IL-22.

AD pathophysiology comprises interactions between the epidermis, immune system, and external environment that collectively determine disease progression [44]. Epidermal malfunction represents the most clinically distinctive characteristic of AD, with breaks in the epidermal layers associated with a Th2-dominant inflammatory response [45]. The most common malfunction in the epithelium is loss-of-function (LOF) mutation in the filaggrin (*FLG*) gene, which greatly increases AD incidence [46, 47]. A recent systematic review showed that the incidence of filaggrin LOF mutations varied with geographic latitude, from 15% of AD patients near the equator to > 25% in the Northern latitudes. Prevalence in the general population ranged from 2.5% near the equator to 7.5% in Northern latitudes [48]. Filaggrin is a protein produced by skin epithelial cells that maintains dermal homeostasis. Filaggrin promotes keratin filament aggregation for keratinocyte-to-corneocyte transition, enhances water retention, and maintains the skin microbiome by limiting *Staphylococcus aureus* growth [49–51]. Interestingly, IL-4 and IL-13 suppress filaggrin expression as well as compensatory tight junction proteins in filaggrin-deficient cells [52, 53]. Despite its role in AD, *FLG* LOF genetic testing is seldom utilized because it does not currently affect treatment [54–56].

Importantly, the Th2 response can promote skin barrier defects through additional mechanisms separate from filaggrin. This includes altering the lipid makeup of the dermal barrier, which contributes to stratum corneum dysfunction [57, 58]. IL-4 and IL-13 can also decrease skin acidity and decrease tight junction protein levels [53, 59, 60].

#### AD Sexual Dimorphism

A global cohort of 6 to 7-year-olds showed varied AD incidence, from 0.9% in India to 22.5% in Ecuador [61]. In addition to variance by country, age- and sex-dependent differences are observed. Disease rates peak between the ages of 0 and 5 and gradually decrease until plateauing post-puberty. AD has no clear sex-based bias in preadolescence. However, after puberty, women have significantly higher rates of AD than men, with variations by region [29, 30, 62–66]. For example, the US CDC reported rates of 8.9% in women versus 5.7% for males in the US (1.5-fold), while Italy showed a female: male difference of 10% versus 6% [67]. In terms of disease severity, US- and Swedish-based studies found that females were not more likely than males to have severe AD [63, 64].

An interesting distinction is found when examining sex differences in the less common intrinsic (nonallergic) form of AD (IAD). These studies are small but show a striking female predominance. A German group showed that 16 of 18 IAD patients were female [68]. Similarly, a Japanese study showed 13 of 17 females among their IAD subjects, while in a Thai study, 6 of 7 were female [69]. Finally, a slightly larger Dutch study identified 34 IAD subjects, of which 70% were female, a rate similar to that in their extrinsic AD cohort [70]. Collectively, these studies found that 59 of 76 IAD subjects were female, a 3:1 ratio.

While AD severity does vary significantly between the sexes, sex hormones affect skin barrier permeability and hence symptomology. Testosterone impairs skin barrier function [71], while estrogen promotes it [72]. Mouse models using ovariectomy to mirror menopause showed decreased skin barrier integrity that correlated with more itch/scratch cycles and was inhibited by blocking IL-4 and IL-13 [73]. Ovariectomized mice also showed increased transdermal water loss correlated with decreased filaggrin and involucrin expression [74]. Skin barrier damage was compensated by estrogen replacement therapy [75]. In contrast, castrated male mice demonstrated increased barrier recovery that was suppressed by testosterone replacement therapy [71]. These mouse models support a common claim in the lay press that menopause worsens AD symptoms, which has not been shown in peer-reviewed studies.

Mast cells are a logical cellular target for studying AD sexual dimorphism. Estrogen has been shown to stimulate mast cell degranulation and enhance IgE-receptor induced degranulation [66, 76, 77]. On a transcriptional level, Mackey et al. observed that mast cells from female mice expressed elevated markers of allergic inflammation relative to their male counterparts. The female mast cells also had higher expression of granule-associated synthesis and storage genes, potentially contributing to higher concentrations of mediators promoting AD [78].

Innate Lymphoid 2 Cells (ILC2) are another cell type potentially driving AD sexual dimorphism. Although not strictly related to AD pathophysiology, previous findings have shown that androgens decrease ILC2 proliferation and IL-5 and IL-13 production in the lungs [79–81]. More recently, Chi et al. found that skin ILC2 cells express high levels of the androgen receptor and that androgens reduced skin ILC2 numbers in mice. In contrast, estrogen promotes dermal DC and Langerhans cell differentiation [82–84]. ILC2s produce granulocyte-macrophage colony-stimulating factor (GM-CSF), which increases skin DC numbers and hence antigen presentation [17]. Thus, estrogen could be promoting ILC2-to-DC communication that enhances Type 2 skin inflammation, while androgens suppress this cascade.

### Allergic Rhinitis (AR)

Characterized by rhinorrhea and nasal congestion, allergic rhinitis (also called hay fever) affects 13 million Americans [85] and > 500 million people worldwide [86, 87]. Global AR rates are highly variable, ranging from 1% to 60% incidence depending on the country [88–90]. Annual costs of AR treatment in the US totaled approximately $4.6 billion in 2022 [85, 91]. A survey conducted in 2012 to determine the deleterious effects of AR in the US, Latin America, and the Asian-Pacific region found that 10% of individuals in the US living with AR experienced absenteeism, in addition to a 33% decrease in productivity among AR patients in all three regions. The study also found that loss of sleep, fatigue, and feelings of depression associated with AR symptoms were commonly reported in all three regions, albeit with varying levels [92].

While rhinitis can have allergic and nonallergic presentations, we will focus on cases presenting with atopic signatures, such as elevated IgE and T2 immune cell infiltration [86]. AR often presents with comorbidities of asthma, atopic dermatitis, or conjunctivitis, due to their overlapping pathophysiologies. However, distinct phenotypes with AR as a primary disease or multimorbidity presentation have made it clear that distinct molecular differences likely exist between these diseases [93, 94]. Allergic rhinitis and its severity are often classified under the Allergic Rhinitis and Its Impact on Asthma (ARIA) scale, marked as intermittent or persistent, and further categorized as either mild or moderate-to-severe based on frequency and severity, respectively. Recurrent survey-type measurement tools such as the AR Visual Analogue Scale (VAS) are also used to quantify disease severity beyond the ARIA grading scale [95, 96].

#### AR Pathophysiology

Allergic rhinitis is a T2 inflammatory disease, involving Th2 cell infiltrate and IgE-mediated hypersensitivity in the nasal mucosa. Damage to the nasal epithelium releases alarmin cytokines (TSLP, IL-25, and IL-33) and leads to allergen sensitization via interactions with resident mucosal antigen-presenting cells. Alarmins directly activate mast cells, basophils, eosinophils, and Type 2 innate lymphoid cells (ILC2), contributing to both the acute and chronic phases of allergic rhinitis [97]. This response also promotes IgE production. IgE crosslinking and alarmin signaling prompts mast cells and basophils to secrete pro-inflammatory mediators including IL-4, IL-13, leukotrienes, and histamine, which collectively elicit AR symptoms.

As stated above, allergic rhinitis is also part of the ‘atopic march’ [98]. There is emerging evidence, however, to suggest that this relatively straightforward model of airway disease characterizes a subtype of allergic disease progression and does not fully explain most allergic rhinitis cases. In 2012, a study analyzing clinical data from nearly 10,000 children in the UK found that only about 3% of the cohort exhibited the atopic march. In fact, over three times as many children studied (9.6%) presented with rhinitis exclusively [99]. This is not to suggest that these allergic conditions are unrelated. It is established that patients experiencing allergic asthma are more likely to develop allergic rhinitis and to present with more severe symptoms – and this relationship is bidirectional [100–104]. In fact, the group conducting the 2012 study published a follow-up in 2019, showing some of the cohort shared genetic profiles, particularly those bearing SNPs in the filaggrin (*FLG*) gene. Among these participants, atopic dermatitis was more strongly associated with comorbid wheezing and allergic rhinitis. The data suggest a relationship between loss of epithelial integrity and the development of allergy aligning with the ‘atopic march’ [98, 105]. In this regard, it is worth noting that many studies address allergic rhinitis as an asthma comorbidity. There appear to be unique molecular pathways differentiating allergic rhinitis from asthma and other atopic diseases.

#### AR Sexual Dimorphism

Differences in allergic rhinitis prevalence between the sexes are modest and follow an age-dependence resembling allergic asthma. A 2017 meta-analysis of 67 studies found that male children under age 11 were 1.2-fold more likely to exhibit rhinitis symptoms than female counterparts. This dichotomy reversed during adolescence (11 to 18 years), with the male frequency dropping to 0.90 compared to females. By adulthood, no sexual dimorphism was found [56]. Single-center and country-specific studies have demonstrated similar results, although some studies have demonstrated a more pronounced female dominance in adults [106–108].

Interestingly, dimorphism appears to be more pronounced when studying the co-occurrence of AR with asthma in children and adolescents. A meta-analysis of 10 studies (> 90k participants) found that pre-adolescent males were 1.65-fold more likely to have both diagnoses than females, a rate that dropped to 0.61 in adolescence. Again, sexual dimorphism was absent in adults when assessing AR and asthma co-occurrence [109].

Thus, it appears that a shift from male-to-female dominance in allergic rhinitis occurs during puberty, indicating the potential role of sex hormones. Why this difference fades in adulthood is unknown, but there is consistent population-level evidence that female sex hormones promote AR incidence. For example, a study of > 40k women from the All of Us project found that menopause is associated with a small but significant decrease in AR incidence, with an adjusted odds ratio (aOR) of 0.89 [110]. These data align with a study showing longer lifetime exposure to estrogens increased AR occurrence, with older age at menopause yielding increased AR incidence (OR = 1.42) [111]. In agreement with the concept that estrogen increases AR, Liu et al. found that hormone replacement therapy (HRT) increased AR selectively in lean women (OR = 2.26) [112]. This group postulates that the association between HRT and body mass index (BMI) is due to HRT reducing insulin resistance among patients with higher BMIs, which should decrease AR. Collectively, these studies support the concept that estrogens, especially longer exposure to hormone effects, increase AR development. This area of study remains relatively unexplored at the cellular level, in part due to the overlapping pathophysiology of AR with asthma. For instance, while it is understood that mast cells, T-cells, dendritic cells, ILC2s, and epithelial cells are affected by estrogens and androgens, these interactions have not been tested in upper airway inflammation models [76, 79, 82, 113].

### Chronic Rhinosinusitis

Chronic rhinosinusitis (CRS) generally presents in two phenotypes – with or without nasal polyps. CRS is characterized by long-term inflammation of the mucosal tissue within the nasal passages and sinuses of the paranasal regions. Nasal endoscopy or diagnostic imaging are typically used to diagnose CRS and associated polyps. The distinction between chronic rhinosinusitis without nasal polyps (CRSsNP) and chronic rhinosinusitis with nasal polyps (CRSwNP) is important because the polyps are eosinophil-rich, emphasizing a Th2-dominant response [114, 115]. These patients also respond well to steroids.

There are several environmental triggers for CRS as well as genetic predispositions. Microbial agents, allergens, and toxins are linked to CRS, and this relationship is augmented when the patient’s immune system is deficient. There are also weaker associations between the onset of CRS and genetic disorders, autoimmune disorders, and immunodeficiency. The literature also indicates that CRSsNP onset may result from bacterial infection that was incompletely treated, while CRSwNP may result from a genetic predisposition to allergic disease [114, 115].

#### CRSsNP pathophysiology, sexual dimorphism, and treatment

While many symptoms are shared between the two CRS phenotypes, CRSsNP tends to present with more prominent facial and ear pain [116]. Importantly, CRSsNP can be characterized by either a Th1 or Th2 immune response. The Th2-dominant endotype has elevated IL-4, IL-5, and IgE [117]. The role of innate immune cells is still being uncovered. When nasal epithelial cells were subjected to hypoxic conditions mimicking CRSsNP, these cells upregulated chemokines and adhesion molecules promoting neutrophil invasion [118]. There is conflicting evidence in the literature regarding the role eosinophils play in CRSsNP, and this needs further investigation [119].

There is a moderate female dominance in CRSsNP [120]. Tan et al.. examined a cohort of patients over 10 years and found 58.2% of patients who were diagnosed with CRSsNP were female (odds ratio of 1.4) [120, 121]. A study utilizing a South Korean national database suggested that symptoms may be worse in females. This group determined that the health-related quality of life of female CRSsNP patients was greatly diminished compared to healthy female volunteers, while no significant association was found with the male patients [122]. Another study found that subjective perception of symptoms was higher in females than in males, even when normalizing for objective disease measures like CT scans [123]. The Tan et al. study suggests that hormones may play a role in CRSsNP. After controlling for age and sex, it was observed that pregnancy was associated with decreased onset of CRSsNP, with an odds ratio of 0.7 [120, 121]. Another study found that post-menopausal CRSsNP patients showed a decrease in symptom severity linked to hormone replacement therapy, suggesting that estrogen deficiency promotes CRSsNP symptoms [124].

#### CRSwNP pathophysiology, sexual dimorphism, and treatment

Nasal polyps are inflammatory lesions that typically appear bilaterally in the sinuses when present. These lesions can project into the airway of the nasal cavity and restrict airflow. Symptoms of CRSwNP usually include nasal congestion, anterior or posterior rhinorrhea, facial pressure or pain, and hyposmia [125]. CRSwNP is often associated with allergic diseases, including asthma, aspirin-exacerbated asthma, and allergic rhinitis, as well as sleep apnea and gastroesophageal reflux disease [121]. Nasal polyps have more recently been characterized as a Th2 immune response marked by eosinophil infiltration. CRSwNP polyps have elevated levels of IL-5 and galectin-10, mediators of eosinophilic inflammation [126], suggesting these cells may be critical to the disease.

Males experience CRSwNP more often than females [115, 125]. Stevens et al.. conducted a retrospective cross-sectional study, which showed that 62% of patients diagnosed with CRSwNP in the cohort were male (1.6-fold). However, this same study determined that females tend to experience more severe CRSwNP than males, much like CRSsNP. 61% of patients reporting severe CRSwNP symptoms were female. Women were twice as likely as men to have radiologic evidence of severe sinus disease [127].

Additional sexual dimorphism noted in CRSwNP is its associations with other medical conditions. One study detailed that 41% of females in its cohort exhibited both asthma and CRSwNP, while only 25% of males exhibited both [128]. Another study observed that female CRSwNP patients were 1.6 times more likely to have asthma and 2.7 times more likely to have allergic rhinitis compared to males [129]. These female-dominant associations warrant more investigation of the underlying mechanisms. Like CRSsNP, estrogen may play a protective role in patients with polyps. Postmenopausal patients who experience CRSwNP and receive hormonal replacement therapy tend not to require surgical intervention for symptom management [124].

### Asthma

Asthma is one of the most common chronic diseases of children and young adults worldwide, accounting for an enormous amount of global healthcare costs and loss in productivity [130]. In the United States alone, the total economic burden of asthma exceeds $90B USD [131].

#### Asthma Pathophysiology

Although there are several manifestations of asthma, allergen-induced allergic asthma constitutes up to 65% of cases [132]. Allergic asthma is largely mediated by an IgE-dependent pathway, causing chronic airway inflammation that limits airflow via bronchoconstriction, edema, mucus hypersecretion, and airway hyperresponsiveness [133]. Once exposed to an allergen, IL-25, IL-33, and thymic stromal lymphopoietin (TSLP) “alarmin” cytokines are released by airway epithelial cells. The alarmins activate dendritic cells and ILC2 cells, which promote Th2 polarization and subsequent Th2-driven eosinophil recruitment, IgE class switching, and mucus hypersecretion [134, 135].

The major immune cells involved in asthma include epithelial cells, smooth muscle cells, macrophages, dendritic cells, neutrophils, eosinophils, lymphocytes, and mast cells [136]. When crosslinked with IgE, mast cells degranulate and cause bronchoconstriction, airway edema, and acute symptom exacerbation [137]. Chronic inflammation causes structural remodeling in the airway, including subepithelial fibrosis, smooth muscle hypertrophy, and increased vascularization [133]. Collectively, these changes create a hyperresponsive airway environment.

#### Sexual Dimorphism in Asthma

Sexual dimorphism is evident in asthma, with striking differences observed in children and adults. Sex differences in childhood are age-dependent and likely influenced by sex hormones during puberty. In young children, males show a ~ 1.5-fold greater prevalence of asthma and greater symptom severity than females. This difference fades until females show a similar 1.5-fold greater prevalence and severity after puberty and into adulthood [138]. Specifically, male-biased prevalence is absent at the mean age of 11.1 years, and female dominance is then noted at the mean age of 16.3 years [139]. Importantly, these studies have examined populations with an asthma diagnosis and are not specific to allergic asthma.

In terms of mechanisms, it is interesting that young males more often exhibit narrow airways relative to lung volume (dysanapsis) [140]. This prompts younger males to experience more asthma symptoms due to narrower airways that grow wider with age. Importantly, the immune cells involved in asthma exhibit sexual dimorphism [17, 78, 141–145]. For example, androgens like testosterone suppress ILC2 proliferation and type 2 cytokine production. Females have higher ILC2 numbers and more Th2-causing inflammation. Estrogen can also increase type 2 inflammatory pathways in macrophages [79, 146].

Not only incidence, but also asthma severity and death are biased towards females in adulthood. Clinical data from the Severe Asthma Network in Italy (SANI) showed that 61.8% of 1,123 patients with severe asthma were female. Women also experienced significantly poorer control, more hospitalizations, and were more likely to present with obesity (22.1% vs. 12.8%) and gastroesophageal reflux (39.9% vs. 28.5%) than men [147]. Classic type 2 inflammation is detected more commonly in males (high IgE levels and eosinophils). Females often present with reduced corticosteroid responsiveness, contributing to poorer control and greater morbidity [147, 148]. Environmental and behavioral factors, including pollutants in the occupational environment and higher rates of pet ownership among women, may further worsen asthma symptoms [147].

Since puberty coincides with the reversal in sexually dimorphic asthma rates, it is reasonable to suggest that sex hormones play an important role. Estrogen, progesterone, and other female hormones active in menstruation, pregnancy, menopause, and hormonal contraceptives have been associated with increased asthma prevalence – particularly when their levels fluctuate. Pre- or perimenstrual asthma affects 11%−45% of women due to hormone fluctuation that worsens symptoms [148]. Women who underwent surgically-induced menopause had an increased risk for asthma onset [149]. Longitudinal data further indicate that the transition into menopause is a significant predictor of new-onset asthma, with the highest risk observed in late postmenopausal women [150]. Beyond the new-onset risk, the menopausal period is also associated with increased asthma severity, a 5-fold higher risk of poor symptom control, and a greater prevalence of comorbidities such as obesity and cardiovascular disease [151]. In contrast, testosterone, dehydroepiandrosterone, and other androgens have been associated with a reduction in asthmatic symptoms [152]. Clinical trials are testing exogenous androgens to limit the severity of asthma symptoms [153, 154].

The most striking demographic differences among asthma patients are found in mortality studies. As reported by the US CDC, asthma mortality increases with age, from 1.6 deaths/million among children ages 0–4 to 29.5/million among adults 65 or older [155]. The asthma death rate for boys (3.1/million) is nearly 50% higher for girls (2.2/million). In contrast, adult women have a death rate of 16.0/million versus men at 10.2/million. Many factors contribute to these differences (e.g., comorbidities among those over 65), but the evidence of sexual dimorphism is clear. While there is research supporting the influential role of sex hormones in asthma, more work is needed to determine mechanisms that may be therapeutically targeted.

### Food Allergy

Food allergy (FA) affects > 10% of adults (at least 26 M Americans) and ~ 8% of children in the United States [156, 157]. It occurs when the immune system produces IgE antibodies against specific food proteins. The characteristics of FA encompass a range of symptoms, including hives, rash, throat edema and pruritus, intestinal distress, and anaphylaxis [158–160]. The symptoms can vary from mild to life-threatening. Importantly, food allergy has increased significantly in the past several decades. For example, the prevalence of food allergy among American children was 3.4% in 1997 and 5.1% in 2011 [40]. Strikingly, peanut allergy among children tripled from 0.4% to 1.4% in a single decade (1997–2008) and has increased significantly among adults as well [161, 162]. This is an area of need for clinical progress.

#### FA Pathophysiology

The skin-gut axis plays an important role in food allergies [163]. This is a bidirectional connection between the skin and gut that involves a network of T cell-mediated immune responses, the microbiome, and systemic signaling [164]. Skin inflammation, as seen in AD, triggers a systemic immune response that affects the gastrointestinal tract, leading to dysbiosis, an imbalance of microbial populations that compromises the intestinal barrier [165, 166]. Inflammatory mediators and immune cells from the intestine migrate to the skin through receptors such as CCR4 via the lymphatics and bloodstream, triggering a cutaneous inflammatory response [167, 168]. Therefore, there is reciprocal migration of T cells and inflammatory mediators between the cutaneous and intestinal tissues, causing a “vicious cycle” [169, 170]. Skin-based allergic sensitization occurs when food allergens cross a disrupted skin barrier, triggering the release of danger signals such as IL-33 from epithelial cells [171]. Cytokines activate antigen-presenting cells, such as dendritic cells, leading to the Th2 response [171]. In clinical studies, patients with AD have a higher risk of developing food allergies, suggesting that the skin is essential for sensitization to food allergens [166]. Leyva-Castillo et al. demonstrated that skin irritation increases intestinal mast cells in the gut, making food allergies worse [172]. In the gut of AD patients, protective bacteria are decreased, and risk-increasing bacteria are increased. This study indicated a genetic link between the gut microbiome and AD through the use of Mendelian Randomization (MR) [173].

#### FA Sexual dimorphism

Following the pattern seen with asthma and AR, males are more likely than females to develop IgE-mediated food allergies in childhood [174], with an estimated 1.8:1 ratio. This trend reverses after puberty, with adult females having a predisposition 1.9-fold greater than males [174, 175]. There is a clinically significant exception to this trend: peanut allergy is twice as common in girls, equally common among men and women ages 30–60, and again twice as common among women over age 60 [162].

Mouse models have been useful for identifying mechanisms underlying food allergy and sexual dimorphism. Wang et al. found that female mice experienced a more severe systemic anaphylaxis than male mice in an ovalbumin-induced FA model [176]. This group found that female mice had high levels of estrogen bound to estrogen receptor β (ERβ), which can induce intestinal inflammation through the PPARγ/NF-κB pathway [176]. While not testing food allergy directly, two additional groups noted stronger anaphylaxis in female mice using a passive IgE-sensitization protocol. Hox et al. found that the female dominance could be traced to estrogen-mediated upregulation of endothelial nitric oxide synthase [177]. Mackey et al. found that mast cells cultured from female mice had more cytoplasmic granules and correspondingly higher levels of histamine, TNF-α, and mast cell proteases [78]. These studies support the hypothesis that female mast cells are inherently more responsive. A role for estrogen is likely, since several studies have shown that estrogen enhances mast cell mediator release [76, 77, 178–180]. Interestingly, there is little literature support for menopause or hormone replacement therapy affecting FA incidence or severity, although associations are commonly noted in popular media. More research is needed.

### Chronic Urticaria

Urticaria is a common disorder affecting 15–25% of the population [181]. The condition is caused largely by mast cell-derived histamine, which elicits itchy wheals – raised, edematous skin lesions. The clinical forms of urticaria are defined by duration and source. Acute spontaneous urticaria is defined as wheals lasting less than six weeks, often induced by food or other allergens. This represents most patients, who usually respond well to standard treatments. Chronic inducible urticaria (CIndU) is defined by the occurrence of wheals or angioedema caused by physical factors (e.g. touch or pressure), with symptoms lasting six weeks or more [182]. Chronic spontaneous urticaria (CSU) consists of wheals and/or angioedema lasting at least six weeks in the absence of an identifiable extrinsic trigger [182, 183]. This has also been referred to as chronic idiopathic urticaria, a term avoided because it can be confused with chronic induced urticaria. CSU and CIndU have spontaneous remission rates of about 50% in 5 years [184]. Many patients require long term treatment.

#### Chronic Urticaria Pathophysiology

CSU affects approximately 0.1–0.3% of the US population and is considered an autoimmune condition in which mast cells are activated by autoantibodies [185]. These include IgG antibodies binding IgE or FcεRI, as well as IgE autoantibodies, often binding thyroperoxidase or IL-24 [186]. A useful, if imperfect, assessment tool is the autologous serum skin test (ASST), which uses intradermal autologous serum injection to elicit wheal formation as a positive result. Up to 50% of CSU patients exhibit a positive ASST [187]. This supports an autoimmune etiology. However, positive ASST outcomes in asymptomatic subjects suggest that autoantibodies are not sufficient for disease occurrence [188, 189]. Moreover, the test is better suited for detecting autoimmune IgG antibodies binding to mast cells than IgE specific for host proteins, as IgE is scarce in the serum [190]. Thus, the ASST can provide both false positive and false negative results and must be interpreted alongside clinical findings. Separate from ASST, several other tests are available and recently reviewed by Larenas-Linnemann [190]. These include measures of basophil activation, for example. However, the autoimmune IgE endotype remains more challenging to detect as it yields a negative result in most assays. In fact, these negative results in the context of clear symptoms, elevated IgE, and comorbid atopy support the diagnosis [190].

CIndU affects approximately 0.5% of the population and primarily results from mast cell activation by physical or environmental factors, leading to histamine release. These patients can be sensitive to low or high temperatures, exercise, and vibration. The most common initiating factor is sustained dermal pressure, which can be exhibited by prominent dermatographism [191]. While the eliciting stimuli are overt, their means of activating mast cells are enigmatic. This is a critical knowledge gap; the symptoms suggest rapid mast cell activation by myriad environmental factors without clear mechanisms.

#### CSU and CIndU Sexual Dimorphism

Chronic urticaria is at least 2-fold more common in females than males [192, 193]. Moreover, women show statistically worse measures of the disease, including: the presence of angioedema, sleep disturbance, systemic symptoms, fatigue and impact on overall quality of life [194–196]. Disease duration has also been shown to be longer in females (33 versus 26 months) [197]. ASST positivity is detected more frequently in females than in males [193]. Interestingly, clinical remission was not sex-dependent in one study [198]. Disease recurrence was also shown by Limphoka et al. to be equal among the sexes [199]. However, recurrence after omalizumab treatment found a 77% recurrence among females and only 36% among males (*p*=.024) [200].

The literature discussing sex hormone effects on chronic urticaria is sparse. Ornek et al. (2023) found among 111 chronic urticaria patients (not distinguishing CSU and CIndU), 83% reported no change in symptoms due to puberty, pregnancy, lactation, or menopause [201]. In contrast, other studies reported that decreased estrogen during menopause can promote mast cell activation, potentially worsening chronic urticaria symptoms [202]. Related to this, a recent study of > 4000 CSU patients found the highest symptom burden among females aged 51–65, a phenotype that diminished after age 65 [194]. This suggests that changes in ovarian hormones can affect CSU symptoms, but clearly more work is needed.

### Systemic Mast Cell Activation Disorders (MCAD)

**Fig. 1:**
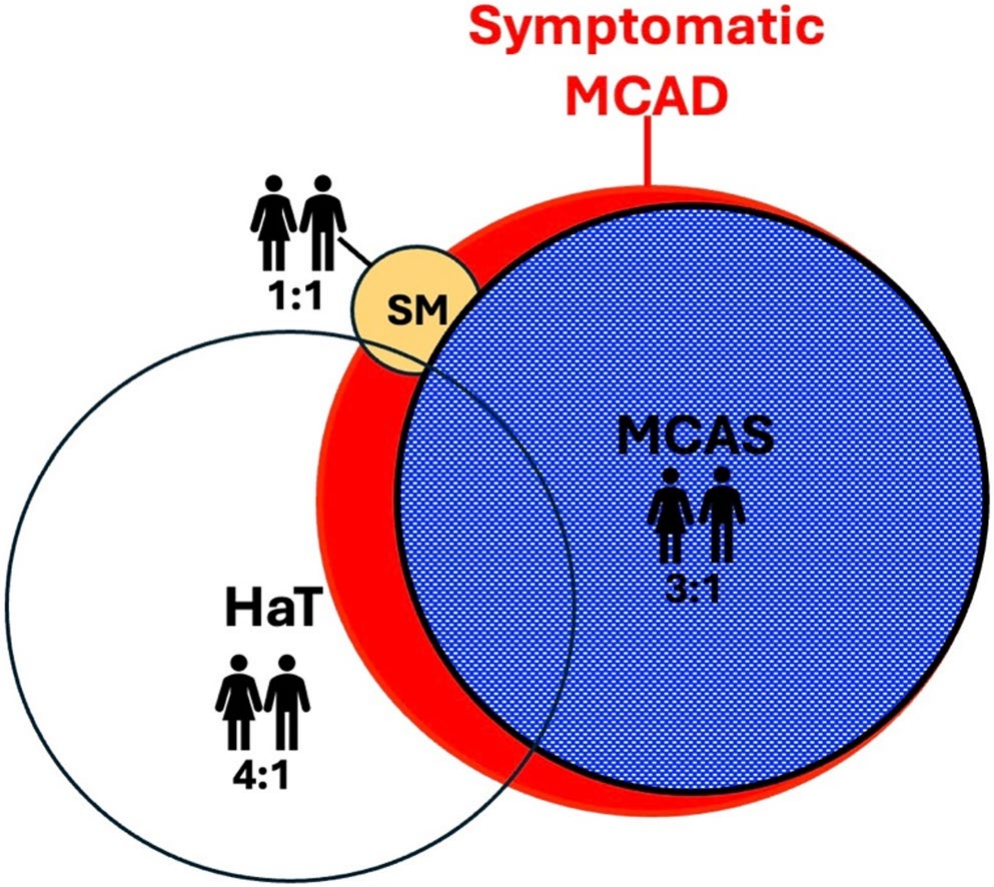
**MCAD etiology and sexual dimorphism** MCAD is defined by symptoms of mast cell mediator effects that do not fulfill a diagnosis of other diseases (e.g., chronic urticaria). The smallest group of MCAD patients has SM, a clonal mast cell expansion. Asymptomatic SM is ill-defined, but not all patients have MCAD symptoms. HaT is a gene amplification found in 5–7% of the general population. About a third of carriers develop MCAD symptoms. The majority of MCAD patients are defined as having MCAS with evidence of mast cell activation in the absence of either mast cell expansion or increased alpha tryptase expression. However, the relative size shown here is based on the wider Consensus 2 criteria. MCAS prevalence is thought to be in single-digit percentages of the general population. As indicated, there is also overlap among the conditions as discussed by Castells et al. [231]. MCAS patients can be subdivided into those with HaT or SM, and SM can also be present in HaT carriers. Females are overrepresented in MCAS and HaT. Importantly, female dominance in HaT is selectively described in those presenting with symptoms; the female: male ratio may be nearly even among asymptomatic carriers, although data are scarce

In 1984, a rare clonal mast cell expansion either limited to the skin or affecting systemic organs was described and subsequently termed cutaneous mastocytosis (CM) or systemic mastocytosis (SM), respectively [203]. Subsequently, symptomatically overlapping disorders were described that do not include increased mast cell numbers (Fig. 1). Collectively, these patients share features attributed to systemic mast cell activation, including flushing, gastrointestinal reflux and/or diarrhea, and syncope. In addition, issues atypical for allergic disease are also noted such as fatigue, fibromyalgia, and neuropsychiatric conditions, including brain fog, anxiety, and depression. This collection of conditions, along with evidence of increased mast cell mediators, is referred to as Mast Cell Activation Disorder (MCAD), emphasizing the role of mediator release rather than mast cell number. Importantly, there is no unified definition of MCAD. For this review, we are using MCAD narrowly, referring to disorders whose symptoms are directly linked to mast cell activation and lack another diagnostic name.

#### Systemic Mastocytosis (SM)

SM is a rare clonal expansion of mast cells, with an incidence of approximately 1–2 patients per 10,000 people. Patients with this myeloproliferative disorder have a high concordance with the KIT D816V mutation and demonstrate a range of disease severity linked to the extent of clonal expansion [204–206]. For example, a recent study of > 3400 SM patients found 50.8% with indolent mastocytosis (IM) that presents most often in the bone marrow, and 31.6% with cutaneous mastocytosis (CM) restricted to the skin. Nearly 20% of patients had systemic mast cell expansion with increasing disease severity, subcategorized as smoldering SM (2%), aggressive SM (3.3%), SM with an associated hematologic malignancy (SM-AHN; 8.3%), and mast cell leukemia (MCL; 1.3%) [207]. As expected, these distinctions greatly impact prognosis, but they also reveal considerable sexual dimorphism.

Among MCAD, SM shows the least female bias, with studies showing an approximate 55:45 ratio favoring females (Table 1). However, sex differences are evident among the subtypes of SM as well as associated pathology. Using Danish Health System records, Broesby-Olsen found that among 687 SM patients, anaphylaxis was unsurprisingly increased relative to the general population (HR = 7.2). However, males showed a 10.1-fold greater likelihood, while the female risk was 5.7-fold. Similarly, the incidence of osteoporosis was 7.1-fold greater among male patients, compared to 2.9-fold for females [204]. Increased osteoporosis among male SM patients was also noted by Zanotti et al., who found that fragility fractures occurred in 12% of males with SM, compared to 2.5% of females [206]. SM organ involvement also varies by sex. Kluin-Nelemans found that females comprised 67% of the “mastocytosis in the skin” subtype, while SM-AHN was 70% male. Partly due to a trend toward more aggressive disease, male SM patients had lower overall survival than females (17.4 years versus 28.4 years, *p*<.0001). A separate study from Lim et al. found that among 342 SM patients, overall survival for males was considerably shorter than females (45 months versus 136 months, *p*=.001) [212]. SM patients generally show little clinical progression toward a more aggressive subtype over time. However, males were more likely to advance than females in the Kluin-Nelemans study (6% versus 3.7%, *p*=.0002). In addition to linking worse outcomes among males to increased disease severity (e.g. SM-AHN subtype), the authors also found that the KIT D816V mutation was slightly more common among males than females (84% versus 75%, *p*<.0001) [207].

**Table 1:** Summary of studies supporting female bias in MCAD-associated diseases

MCAS Studies	% Female	Total *N*	SM Studies	% Female	Total *N*	HaT Studies	% Female	Total *N* (HaT+)
*Häder et al.*,* 2023* Defined by Consensus 2 criteria [208]	**86%**	250	*Kluin-Nelemans et al.*,[207]	**55.3%**	3407	*Puxkandl et al.*,* 2024* Patients with elevated serum tryptase compared to control group with history of anaphylaxis after bee sting [209]	**86%**	44
			*Ungerstedt et al.*,[210]	**55.7**	195			
*Novak et al.*,* 2022* Defined by Consensus 1 criteria [211]	**87.5%**	16	*Lim et al.*,[212]	**45%**	342	*Robey et al.*,* 2020* PCR screen of 432 unselected samples from birth registry, identifying 70 with HaT [213]	**64%**	70
*Hamilton et al.*,* 2021* Defined by Consensus 1 criteria [214]	**93.3**	15	*Cohen et al.*,[215]	**60%**	548			
*Molderings et al.*,* 2017* Defined by Consensus 2 criteria [216]	**74% (Germany)** **70% (USA)**	828	*Bergstrom et al.*,[205]	**60%**	1040	*Giannetti et al.*,* 2021* 101 patients referred for with MCAS symptoms (81% female) [217]	**80%**	101
			* Broesby-Olsen et al.*, [204]	**60%**	687	*Chollet and Akin*,* 2022* 38 subjects recruited from allergy clinic or biorepository [218]	**79%**	38
*Molderings et al.*,* 2013* Defined by Consensus 2 criteria [219]	**74%**	84						
*Afrin et al.*,* 2017* Defined by Consensus 2 criteria [220]	**69%**	413						

Collectively, we found 6 studies reporting SM outcomes by sex (Table 1). Of these, 5 show a modest female bias for disease incidence. In contrast, increased disease severity, comorbidities, and lower overall survival were noted among male patients. It is worth noting that the Kluin-Nelemans study found that among 190 SM patients with advanced disease (2:1 ratio favoring males), multiple high-risk mutations distinct from KIT D816V were more common among males than females (88% versus 70%, *p*=.004) [207]. It is plausible that males have more exposure to mutagenic factors than females, increasing the risk of advanced SM. However, the literature also suggests that SM is inhibited by factors in the female cellular environment, exacerbated by male factors, or perhaps both.

#### Hereditary Alpha Tryptasemia (HaT)

Two reports of patients presenting with allergy symptoms and high basal serum tryptase (BST) levels in the absence of SM were published in 2014 [221, 222]. These cases showed Mendelian inheritance, which was subsequently explained by increased copy number of the alpha tryptase gene (TPSAB1). Subsequently termed Hereditary alpha tryptasemia (HaT) [223], the condition is enigmatic for several reasons. First, it is a genetic trait, not a syndrome, as less than one-third of HaT individuals present with symptoms [213, 223, 224]. Further, monoallelic diseases are rare, but HaT is surprisingly common, found in 5–7% of the White, Western population [213, 218, 225, 226]. Further, at least one study found that alpha tryptase is often deleted in the general population, with 29% of individuals lacking the gene (*N* = 274). This group also noted a racial/geographic effect: gene deletion occurred in 45% of Caucasians, 26% of African Americans, and was rare among Asians [227]. Finally, alpha tryptase is essentially enzymatically inactive. Instead, it allows beta tryptase to cleave new targets when the two proteins form a tetramer [228]. Collectively, these data yield an evolutionary mystery: alpha tryptase appears to be commonly deleted without effect but also multiplied with only occasional consequence, and both events are more common among the White/Western population.

It is worth noting that HaT subjects often present with additional clinical symptoms. HaT does not appear to increase the incidence of anaphylaxis, but these individuals are twice as common among *severe* anaphylaxis patients as in the general population [229]. This suggests that a HaT genotype worsens anaphylactic reactions. Individuals also show a high incidence of vibratory urticaria [228] and retained primary dentition [230].

While an explanation for this interesting evolution is lacking, one conclusion is that alpha tryptase amplification alone does not cause disease. In support of this, Chollet and Akin identified 31 HaT carriers from 416 biorepository samples. They then compared 10 HaT carriers to 24 controls and found no increase in the prevalence of asthma, rhinitis, anxiety, or depression [218]. Thus, HaT may amplify symptoms without increasing disease prevalence. This is plausible based on novel protease targets when alpha tryptase is partnered with the beta isoform. For example, Le et al. showed that α2β2 heterotetramers can cleave the protease-activated receptor (PAR)−2, exacerbating hypotension, pruritus, and asthma. They also noted cleavage of the alpha subunit of the EGF-like module–containing mucin-like hormone receptor-like 2 (EMR2) mechanosensory receptor, leading to vibratory urticaria [228].

A second, non-exclusive hypothesis is that HaT carriers develop symptoms when augmenting factors are present. This second proposition could be supported by clear evidence of sexual dimorphism: nearly 80% of *symptomatic* HaT patients are female (Table 1). It is unclear if female dominance is present in asymptomatic HaT carriers. One could propose that female sex hormones or other biological factors, coupled with novel tryptase cleavage products, make female HaT carriers more prone to MCAD symptoms.

Whatever the mechanism, symptomatic HaT patients make up a considerable proportion of MCAD cases. It is important to note that disentangling HaT from MCAS might appear simple, using genotyping as a definitive assay. But symptomatic HaT carriers often present with systemic mast cell activation and are proposed as one subtype of MCAS [231]. This can be useful, since HaT suggests one possible etiology for MCAS, where a lack of clear biomarkers and causes has led MCAS patients to suffer for > 4 years on average before diagnosis [232]. Additionally, HaT carriers are 2-fold more common among SM patients, where HaT prevalence is 12–18%, as recently reviewed by Bonadonna et al. [233].

#### Mast Cell Activation Syndrome (MCAS)

The term “mast cell activation syndrome” was first used in a case report in 2007 [234] but patients with evidence of systemic mast cell degranulation in the absence of increased mast cell number were first discussed in 1991 [235]. The literature has grown considerably since MCAS was named, but the disease remains unclear in many respects.

Importantly, there is debate about the MCAS diagnostic criteria. A working group offered diagnostic criteria in 2012, which includes acute clinical symptoms of mast cell activation in at least 2 organ systems, elevated levels of mast cell mediators, and clinical response to mast cell-targeting agents [236]. These criteria were further elaborated in 2019 [237], and a recent paper describes the logic behind these clinical criteria in detail [238]. Serum tryptase is the most accepted marker, with MCAS partly defined as a serum tryptase 1.2-fold higher than baseline + 2 ng/ml, determined within 6 h of symptom onset, as recently detailed by Castells et al. [231]. This relatively uncommon test and the limited time frame contribute to the belief that MCAS is underdiagnosed. Others have offered a second set of diagnostic criteria, which they refer to as “Consensus 2” (with “Consensus 1” being those discussed by Castells et al.). They suggest that serum chromagranin A, plasma heparin, combined with urinary N-methylhistamine and PGF-alpha2, provide fewer false negatives than serum tryptase [216, 220, 239]. Separately, Butterfield and Taylor recently argued that urinary LTE4 may be the most robust clinical measure [240]. These differences matter, because the broader Consensus 2 criteria greatly increase the potential number of MCAS patients, which is estimated in the single-digit percentages [219], although this is also debated [241]. Importantly, a recent study employing large language models found that the broader MCAS diagnostic criteria led to lower diagnostic consistency and precision [242].

One of the few consistent findings in the MCAS literature is a clear female dominance, with an approximate 3:1 ratio, whether the subjects are defined by Consensus 1 or 2 criteria (Table 1). The reason for this sexual dimorphism remains unknown. Two case reports have suggested that the onset of menstruation coincided with anaphylaxis in patients whose clinical symptoms fit MCAD and perhaps MCAS [243, 244]. These offer modest insight, though a role for reproductive hormones in mast cell activation and increased mast cell mediator content of female mast cells [245] could both be implicated.

A separate issue that illustrates the debate and importance of MCAS diagnostic criteria is the possible association of POTS and Ehlers-Danlos Syndrome (EDS) with MCAS. As discussed in detail by Farley et al., there have been multiple reports citing an increased incidence of POTS or EDS with MCAS. When this group performed a systematic review of 92 articles and applied the stricter Consensus 1 MCAS diagnostic criteria, no association was found with POTS or EDS [246]. This is notable for our review because sexual dimorphism among POTS and EDS patients resembles MCAS. POTS has a female bias of 4:1 and increased symptom severity among females [247, 248]. Similarly, EDS patients are approximately 70% female [249]. The female bias may be coincidental but adds to the perceived association between POTS, EDS, and MCAS and emphasizes the importance of a consensus definition of MCAS.

In summary, MCAD is defined by the primary feature of systemic mast cell activation. The major subset of these patients appears to fit the diagnosis of MCAS, though this number is greatly enlarged by the looser Concensus-2 criteria. A significant portion of MCAD patients are symptomatic HaT carriers, and a small fraction have the rare disease SM. Importantly, symptomatic HaT and SM have been suggested to be a subset of MCAS. This generates confusion among non-experts. Despite this, one consistent thread is increased female incidence, with 75–80% of HaT and MCAS patients being female. An important caveat to this sexual dimorphism is that while SM shows little sexual dimorphism in prevalence, males have greater morbidity and mortality. This is strikingly different from HaT and MCAS, where symptoms appear to be worse among females. Finding the mechanism for these sex differences could reveal important aspects of these diseases and should be a primary focus of this field.

### Eosinophilic Esophagitis

As the name suggests, eosinophilic esophagitis (EoE) is a chronic, immune-mediated disorder characterized by eosinophil-dominant inflammation of the esophagus. It has emerged as a leading cause of dysphagia and food impaction in pediatric and adult populations. EoE stands out among allergic diseases by a 3:1 male-to-female predominance. It is increasingly recognized as a late component of the atopic march. EoE is driven by a complex interplay of genetic susceptibility, environmental triggers, and dysregulated Th2-mediated immune responses [250, 251]. In addition to dysphagia and food impaction, primary symptoms include failure to thrive, vomiting, and chest and/or abdominal pain [252]. EoE is rare but increasing in incidence, with an estimated prevalence of 56.7/100,000 individuals in the United States. 52.8% of EoE patients have a diagnosis of at least one other allergic condition. The most affected age group is adults (aged 18–65 years), comprising 74.5% of the cases, while children (aged < 18 years) make up 15.9% of cases, and older adults (aged > 65 years) comprise 9.6% [251, 253]. EoE diagnoses have been increasing partly due to improved recognition amongst the pediatric population, which was largely neglected in endoscopic procedures before the mid-1980s [252]. Heritability risk in EoE is significant. While only about 2% of EoE patients have an affected sibling, the increased relative risk among first-degree relatives is 10–64-fold, and highest among males [251, 254–256].

#### EoE Pathophysiology

EoE pathophysiology is intricate and reminiscent of atopic dermatitis. Initially, the esophageal epithelium, also known as the esophageal barrier, is impaired due to genetic factors. These include diminished expression of filaggrin (FLG), Desmoglein-1 (DSG1), or the serine peptidase inhibitor kazal types 5 and 7 (SPINK5 and SPINK7, respectively). Patients also overexpress eotaxin-3 (CCL26) and calpain-14 (CAPN14) [253, 257, 258]. FLG loss impairs keratinocyte differentiation, compromising barrier function. Loss of DSG1 reduces epithelial cell anchoring, a problem exacerbated by CAPN14-mediated degradation. This results in fluid accumulation beneath the epithelium and cell detachment, also known as spongiosis [259]. IL-13, a key type 2 cytokine, increases CAPN14 while inhibiting DSG1 expression [260]. SPINK 5 and 7 loss also reduces barrier integrity, resulting in intracellular dilations, paracellular permeability, and disruption of adherens junction proteins, including DSG1 [261]. Collectively, these alterations reduce the tight junctions between epithelial cells and increase the permeability of small molecules through the esophageal barrier [262].

Loss of barrier function allows proteins from foods, microbes, and allergens to enter the esophageal barrier as they descend through the lumen [258]. After permeating the epithelium, proteins interact with Langerhans cells, allowing antigen presentation [263, 264]. Some antigens also elicit the release of IL-33, IL-25, and TSLP from epithelial keratinocytes. These alarmin cytokines stimulate ILC2, invariant natural killer T cells (iNKT), and peripheral DC, promoting Th2 polarization [265].

IL-13, produced by mast cells, ILC2, and Th2 cells, increases eotaxin-3 release. These same cells also produce IL-5. Eotaxin-3 and IL-5 guide eosinophils into the lamina propria and muscularis mucosae of the esophagus, where they secrete eosinophilic peroxidase (EPO), eosinophilic cationic proteins, and Major Basic Protein (MBP) [263, 264]. These toxic proteins cause direct cell damage, esophageal dysfunction, and increase smooth muscle cell contractility [264]. As the eosinophils move into the lamina propria, they release transforming growth factor (TGFβ1) alongside Th17 and mast cells, stimulating fibroblast proliferation [260, 264, 266]. This damage and remodeling leads to fibrosis and esophageal strictures, hallmark features of the tissue remodeling found in EoE [251].

Mast cells and basophils are also activated by alarmins and by Th2-driven IgE production [262, 267, 268]. These innate immune cells reinforce the T2 inflammatory response and tissue remodeling. TSLP signaling on basophils elicits T2 cytokine production that supports the Th2 response [266]. Mast cells release prostaglandin D_2_ (PGD2), which binds CRTH2 (chemoattractant receptor-homologous molecule expressed on Th2 cells) expressed on Th2, basophils, and eosinophils.

#### EoE Sexual Dimorphism

A recent review by Mona and Hruz summarized several meta-analyses showing that EoE is most often diagnosed between ages 30–50 and exhibits a 3:1 male bias [269]. Not only are males predominant in the EoE population, but they are also more likely to be diagnosed as children [270], have a longer diagnostic delay than women, and present with more advanced disease [271]. Men and women also present with distinct symptoms. In a study of 162 EoE patients (56% male), Lynch et al. found that women more often presented with inflammatory symptoms, including chest pain and heartburn, while men more frequently reported symptoms related to tissue remodeling and dysfunction, such as dysphagia and food impaction [272]. This finding was backed by similar results from Sperry et al. [270]

Interestingly, the Lynch et al. and Sperry et al. studies found that endoscopic characteristics did not differ between males and females. Similarly, Moawad et al. found that male and female EoE patients (*n* = 793 adults and children, 72% male) had no differences in endoscopic or histologic findings – but males were 50% more likely to have esophageal strictures than females [273]. However, a 2017 study of adult subjects found numerous differences in male and female biopsies [274]. Importantly, this study employed subjects with similar endoscopic characteristics and collected samples before any specific therapy was begun. Specifically, the chemokines CCL24 and CCL26, arachidonic acid lipoxygenase 15 (ALOX15), tryptase alpha/beta 1 *(TPSAB1*), histidine decarboxylase (*HDC*), and SCF/KIT ligand (*KITLG*) were increased in male EoE patients compared to females [274]. *CCL24*,* CCL26*, and *ALOX15* are prominent eosinophil-associated genes. CCL24 and CCL26 bind to the CCR3 receptor, stimulating eosinophil movement and enhancing the Th2 immune response [275]. ALOX15 catalyzes fatty acid oxidation and increases endothelial cell permeability [276]. Tryptase is a hallmark gene for mast cells, whose differentiation and survival are maintained by SCF.

These differences in symptomology and gene expression support the hypothesis that EoE is distinct in males and females, but little else is clear. If the gene signatures support a more robust mast cell disease in males, why do the endoscopic and histologic findings show no striking differences in these studies? Do these differences promote strictures in males or is this only correlative? Does the disease differ significantly between children and adults, and if so, are androgens driving the gene alterations in males? We are left with more questions than answers.

### Allergic Conjunctivitis

Allergic conjunctivitis (AC) is a common immunological disease that inflames the conjunctiva, leading to significant eye discomfort. The economic burden of AC is substantial, with annual treatment costs estimated at $800 million in the United States alone. Furthermore, the indirect costs of lost productivity due to work absences approximate $1.9 billion [277]. Given that AC has a lifetime incidence of perhaps 40% of the adult population, it poses a significant public health concern [278, 279].

#### Allergic Conjunctivitis Pathophysiology

Among several AC variants, perennial allergic conjunctivitis (PAC) and seasonal allergic conjunctivitis (SAC) are the most common. While SAC symptoms typically arise during the spring and summer due to seasonal allergies, PAC occurs year-round, triggered by persistent allergens. Common symptoms across both types include itching, redness, excessive tearing, burning sensation, and swelling of the conjunctiva [280]. Vernal keratoconjunctivitis (VKC) is a more severe form of AC that is most common in tropical or warm climates and primarily affects school-aged children. VKC is a subset of AC and is distinguished by frequent involvement of the cornea, which is unusual in AC. Left untreated, corneal ulcers can form, causing vision to be significantly impaired. General symptoms of VKC include photophobia, mucus discharge, and itching [281]. Atopic keratoconjunctivitis (AKC) is a chronic variant of AC that overlaps with VKC but is more prominent in adults with atopic dermatitis (AD) and can occur year-round. Due to the inflammatory symptoms associated with AD, eyelids in patients with AKC tend to be thickened and hardened, along with impaired meibomian gland function, which irritates the lesion [282].

The conjunctiva is profoundly vascularized and is in direct contact with the environment, making it highly susceptible to airborne allergens such as pollen, dust mites, and mold. Allergic conjunctivitis manifests as a Type 1 hypersensitivity reaction mediated by IgE antibodies bound to FcεRI on the surface of mast cells. These IgE molecules are cross-linked by allergens, leading to histamine and protease release [283]. Among the many ways in which AC can progress, it has been demonstrated that epithelial cells of the conjunctiva secrete IL-33. This augments T2 cytokine secretion (IL-4, IL-5, IL-13) by tissue mast cells, basophils, and ILC2s, further amplifying AC in a late-phase response [284, 285]. Additionally, VKC is associated with significantly higher concentrations of histamine and tryptase in conjunctival epithelium compared to the AC response, indicating its clinical severity [286].

There is an important distinction between AC, AKC, and VKC. While all are strongly associated with allergy, VKC has been found in several studies to present with autoantibodies. Specifically, VKC is associated with anti-thyroid autoantibodies, found in 5%−20% of patients [287], compared to perhaps 0.5% of controls [288]. Also, anti-nuclear antibodies were found in 30% of pediatric VKC patients in another study [289], which is twice the expected rate [290]. Autoantibodies tended to be associated with worse VKC measures and a family history of autoimmunity. Thus, VKC– at least in some patients – may be a fundamentally distinct disease.

#### Allergic Conjunctivitis Sexual Dimorphism

Allergic conjunctivitis (SAC and PAC) epidemiology, at least with respect to sexual dimorphism, is surprisingly scant in the literature. However, there is support for a female bias. Yamana et al. recently reported that among nearly 3000 patients with allergic conjunctival disease seen in their clinic over a 4.5-year period (ages 11–85), females were more common with a 2:1 ratio [291]. Another study of nearly 500 adults found a similar female: male ratio that did not reach statistical significance [292]. A smaller study of 236 patients found that the subset with AC was female-dominant [293]. Finally, a 2015 study of > 1500 pediatric AC patients from Brazil found 56% females (*p*=.01).

Unlike AC, VKC shows a clear *male-dominance*, with a ratio of 4:1 among pediatric patients. This ratio decreases in late-onset adults (which were < 10% of study participants), but males still predominate at 2:1 [294] or 3:1 [295]. Furthermore, in a study evaluating the frequency of estrogen and progesterone receptors on conjunctival cells in VKC patients, 70% of the cells staining positive were eosinophils, whereas a healthy conjunctiva showed no estrogen or progesterone receptor staining [296]. The same group later showed that serum estrogen was elevated in VKC patients, while testosterone was decreased relative to controls [297]. One hypothesis is that male sex hormones are protective, such that elevated androgens in males after puberty diminish VKC incidence.

AKC does not show overt sexual dimorphism. A recent prospective study of > 1000 patients with allergic ocular disease found an even male-female AKC distribution, while 88% of VKC patients were male [298]. It is interesting to note that VKC peaked in the teen years, while AKC diagnosis peaked at age 21–30. Overall, the incidence and pathophysiology of AKC suggest a more severe disease that is consistently linked to allergic inflammation. VKC appears to be an overlapping entity with allergic inflammation that is amplified by more complex mechanisms tied to autoimmunity.

## Conclusion

Many T2 inflammatory diseases exhibit sexual dimorphism. These differences are not fully understood, but the inverting of dimorphism at puberty and menopause-associated shifts support the potent effects of androgens and ovarian hormones. Unraveling mechanisms driving sexual dimorphism could lead to tailored treatments. Perhaps the most important examples of this are allergic asthma, chronic rhinosinusitis, and chronic urticaria - diseases affecting large portions of the population where both incidence and severity exhibit sex-dependence and molecular mechanisms are well-described. These stand in contrast to MCAS, where a female bias is evident but mechanistic insight is lacking. In sum, sexual dimorphism can provide both basic insight into these diseases and possibilities for new therapy. (Table 2).

**Table 2:** A description of sexual dimorphism in allergic diseases

Allergic Disease	Sexual Dimorphism (relative ratio)			
Asthma	Incidence & Severity	Prepubescense	♂ Male-dominant (1.5:1)	
		Postpubescence	♀ Female-dominant (1.5:1)	
Allergic Rhinitis	No Comorbidities		No Sexual Dimorphism	
	Comorbid with asthma		Prepubescense	♂ Male-dominant (1.6:1)
			Postpubescence	♀ Female-dominant (1.6:1)
Food Allergy	Childhood		♂ Male-dominant (1.8:1)	
	Adulthood		♀ Female-dominant (1.9:1)	
Chronic Rhinosinusitis	CRSsNP	Incidence	♀ Female-dominant (1.4:1)	
		Severity	♀ Female-dominant	
	CRSwNP	Incidence	♂ Male-dominant (1.6:1)	
		Severity	♀ Female-dominant	
Atopic Dermatitis	Childhood		No Sexual Dimorphism	
	Adulthood		♀ Female-dominant (1.5:1)	
Chronic Urticaria	Incidence		♀ Female-dominant (2:1)	
	Severity		♀ Female-dominant	
Systemic Mast Cell Activation Disease	SM		Incidence	♀ Female-dominant (1.5:1)
			Severity	♂ Male-dominant
	HaT (Symptomatic)		♀ Female-dominant (4:1)	
	MCAS		♀ Female-dominant (3:1)	
Eosinophilic Esophagitis	Incidence		♂ Male-dominant (3:1)	
	Severity		♂ Male-dominant for esophageal strictures	
Allergic Conjunctivitis	SAC and PAC		♀ Female-dominant (2:1)	
	VKC		Childhood	♂ Male-dominant (4:1)
			Adulthood	♂ Male-dominant (> 2:1)
	AKC		No sexual dimorphism	

## Data Availability

No datasets were generated or analysed during the current study.

## References

[1] Lindsay WR, Webster MS, Varian CW, Schwabl H (2009) Plumage colour acquisition and behaviour are associated with androgens in a phenotypically plastic tropical bird. Anim Behav 77(6):1525–1532. 10.1016/j.anbehav.2009.02.027

[2] Purves D et al (2001) Hormonal Influences on Sexual Dimorphism, in *Neuroscience. 2nd edition*, Sinauer Associates, Accessed: Jun. 12, 2025. [Online]. Available: https://www.ncbi.nlm.nih.gov/books/NBK11161/

[3] Yung JA, Fuseini H, Newcomb DC (2018) Hormones, sex, and asthma. Ann Allergy Asthma Immunol 120(5):488–494. 10.1016/j.anai.2018.01.016 29410216 10.1016/j.anai.2018.01.016 PMC5936670

[4] Maret A et al (2003) Estradiol enhances primary antigen-specific CD4 T cell responses and Th1 development in vivo. Essential role of estrogen receptor alpha expression in hematopoietic cells. Eur J Immunol 33(2): 512–521. 10.1002/immu.200310027 10.1002/immu.200310027 12645950

[5] Cai Y, Zhou J, Webb DC (2012) Estrogen stimulates Th2 cytokine production and regulates the compartmentalisation of eosinophils during allergen challenge in a mouse model of asthma. Int Arch Allergy Immunol 158(3):252–260. 10.1159/000331437 22398379 10.1159/000331437

[6] Mantalaris A et al (Mar. 2001) Localization of androgen receptor expression in human bone marrow. J Pathol 193(3):361–366. 10.1002/1096-9896(0000)9999:9999<::AID-PATH803>3.0.CO;2-W 11241417

[7] Chen W et al (2010) Human mast cells express androgen receptors but treatment with testosterone exerts no influence on IgE-independent mast cell degranulation elicited by neuromuscular blocking agents. Exp Dermatol 19(3):302–304. 10.1111/j.1600-0625.2009.00969.x 19758318 10.1111/j.1600-0625.2009.00969.x

[8] Kissick HT et al (2014) Androgens alter T-cell immunity by inhibiting T-helper 1 differentiation. Proc Natl Acad Sci U S A 111(27):9887–9892. 10.1073/pnas.1402468111 24958858 10.1073/pnas.1402468111 PMC4103356

[9] Aguado BA et al (2022) Genes that escape X chromosome inactivation modulate sex differences in valve myofibroblasts. Circulation 145(7):513–530. 10.1161/CIRCULATIONAHA.121.054108 35000411 10.1161/CIRCULATIONAHA.121.054108 PMC8844107

[10] Carrel L, Willard HF (2005) X-inactivation profile reveals extensive variability in X-linked gene expression in females. Nature 434(7031):400–404. 10.1038/nature03479 15772666 10.1038/nature03479

[11] Souyris M et al (2018) TLR7 escapes X chromosome inactivation in immune cells. Sci Immunol 3:eaap8855 (19). 10.1126/sciimmunol.aap8855 29374079

[12] Youness A et al (2023) TLR8 escapes X chromosome inactivation in human monocytes and CD4 + T cells. Biol Sex Differ 14:60. 10.1186/s13293-023-00544-5 10.1186/s13293-023-00544-5 PMC10506212 37723501

[13] Chen J et al (2024) KDM5D histone demethylase mediates p38α inactivation via its enzymatic activity to inhibit cancer progression. Proc Natl Acad Sci 121(50): e2402022121. 10.1073/pnas.2402022121 10.1073/pnas.2402022121 PMC11648606 39636854

[14] Cunningham CM et al (2022) Y-Chromosome Gene, Uty, Protects Against Pulmonary Hypertension by Reducing Proinflammatory Chemokines. Am J Respir Crit Care Med 206(2): 186–196. 10.1164/rccm.202110-2309OC 10.1164/rccm.202110-2309OC PMC9887415 35504005

[15] Dong J et al (2022) SRY is a Key Mediator of Sexual Dimorphism in Hepatic Ischemia/Reperfusion Injury. Ann Surg 276(2): 345–356. 10.1097/SLA.0000000000004422 10.1097/SLA.0000000000004422 33086308

[16] Dou DR et al (2024) Xist ribonucleoproteins promote female sex-biased autoimmunity. Cell 187(3):733-749.e16. 10.1016/j.cell.2023.12.037 38306984 10.1016/j.cell.2023.12.037 PMC10949934

[17] Chi L et al (2024) Sexual dimorphism in skin immunity is mediated by androgen-ILC2-dendritic cell axis. Science 384(6692):eadk6200. 10.1126/science.adk6200 10.1126/science.adk6200 PMC12086714 38574174

[18] Akdis CA et al (2020) Type 2 immunity in the skin and lungs. Allergy 75(7):1582–1605. 10.1111/all.14318 10.1111/all.14318 32319104

[19] Peckham H et al (2025) Estrogen influences class-switched memory B cell frequency only in humans with two X chromosomes. J Exp Med 222(4):e20241253. 10.1084/jem.20241253 40049222 10.1084/jem.20241253 PMC11893172

[20] Maspero J et al (2022) Type 2 inflammation in asthma and other airway diseases. ERJ Open Res 8(3):00576–02021. 10.1183/23120541.00576-2021 35923421 10.1183/23120541.00576-2021 PMC9339769

[21] Leung DYM, Facheris P, Veverka KA, Cevikbas F, Guttman-Yassky E (2024) Targeting type 2 immune activation beyond atopic dermatitis. Ann Allergy Asthma Immunol 132(2):121–123. 10.1016/j.anai.2023.06.028 37442541 10.1016/j.anai.2023.06.028

[22] Kwatra SG, Ständer S, Yosipovitch G, Kim BS, Levit NA, O’Malley JT (2025) Pathophysiology of prurigo nodularis: neuroimmune dysregulation and the role of type 2 inflammation. J Invest Dermatol 145(2):249–256. 10.1016/j.jid.2024.06.1276 39217537 10.1016/j.jid.2024.06.1276

[23] Sahiner UM et al (2021) Innate lymphoid cells: The missing part of a puzzle in food allergy. Allergy 76(7):2002–2016. 10.1111/all.14776 10.1111/all.14776 33583026

[24] Asada Y (2020) Roles of type 2 immune response-initiating cytokines and detection of type 2 innate lymphoid cells in mouse models of allergic conjunctivitis. Cornea 39(1):S47–S50. 10.1097/ICO.0000000000002548 33038152 10.1097/ICO.0000000000002548

[25] Pesqué D et al (2023) Autoimmune diseases and low baseline IgE in chronic spontaneous urticaria: a clinical and therapeutic prospective analysis in real-life clinical practice. J Allergy Clin Immunol Pract 11(12):3763-3771.e5. 10.1016/j.jaip.2023.09.002 37716526 10.1016/j.jaip.2023.09.002

[26] Boehm T, Ristl R, Mühlbacher J, Valent P, Wahrmann M, Jilma B (2022) Massive release of TH2 cytokines induced a cytokine storm during a severe mast cell activation event in a patient with indolent systemic mastocytosis. J Allergy Clin Immunol 150(2):406-414.e16. 10.1016/j.jaci.2022.04.023 35504498 10.1016/j.jaci.2022.04.023

[27] Menard-Katcher P, Marks KL, Liacouras CA, Spergel JM, Yang Y-X, Falk GW (2013) The natural history of eosinophilic oesophagitis in the transition from childhood to adulthood. Aliment Pharmacol Ther 37(1):114–121. 10.1111/apt.12119 23121227 10.1111/apt.12119

[28] Ahn C, Huang W (2024) Clinical presentation of atopic dermatitis. Adv Exp Med Biol 1447:37–44. 10.1007/978-3-031-54513-9_4 38724782 10.1007/978-3-031-54513-9_4

[29] Tian J et al (2024) Global epidemiology of atopic dermatitis: a comprehensive systematic analysis and modelling study. Br J Dermatol 190(1):55–61. 10.1093/bjd/ljad339 10.1093/bjd/ljad339 37705227

[30] Shin YH et al (2023) Global, regional, and national burden of allergic disorders and their risk factors in 204 countries and territories, from 1990 to 2019: a systematic analysis for the Global Burden of Disease Study 2019. Allergy 78(8):2232–2254. 10.1111/all.15807 37431853 10.1111/all.15807 PMC10529296

[31] Sroka-Tomaszewska J, Trzeciak M (2021) Molecular mechanisms of atopic dermatitis pathogenesis. Int J Mol Sci 22(8):4130. 10.3390/ijms22084130 33923629 10.3390/ijms22084130 PMC8074061

[32] Afshari M, Kolackova M, Rosecka M, Čelakovská J, Krejsek J (2024) Unraveling the skin; a comprehensive review of atopic dermatitis, current understanding, and approaches. Front Immunol 15:1361005. 10.3389/fimmu.2024.1361005 38500882 10.3389/fimmu.2024.1361005 PMC10944924

[33] Czarnowicki T, Krueger JG, Guttman-Yassky E (2017) Novel concepts of prevention and treatment of atopic dermatitis through barrier and immune manipulations with implications for the atopic march. J Allergy Clin Immunol 139(6):1723–1734. 10.1016/j.jaci.2017.04.004 28583445 10.1016/j.jaci.2017.04.004

[34] Gooderham MJ et al (2018) Approach to the Assessment and Management of Adult Patients With Atopic Dermatitis: A Consensus Document. J Cutan Med Surg 22(1) 3S-5S. 10.1177/1203475418803627 10.1177/1203475418803627 30439298

[35] Boguniewicz M, Fonacier L, Guttman-Yassky E, Ong PY, Silverberg J, Farrar JR (2018) Atopic dermatitis yardstick: practical recommendations for an evolving therapeutic landscape. Ann Allergy Asthma Immunol 120(1):10-22.e2. 10.1016/j.anai.2017.10.039 29273118 10.1016/j.anai.2017.10.039

[36] Fishbein AB, Silverberg JI, Wilson EJ, Ong PY (2020) Update on atopic dermatitis: diagnosis, severity assessment, and treatment selection. J Allergy Clin Immunol Pract 8(1):91–101. 10.1016/j.jaip.2019.06.044 31474543 10.1016/j.jaip.2019.06.044 PMC7395647

[37] Furue M et al (2017) Atopic dermatitis: immune deviation, barrier dysfunction, IgE autoreactivity and new therapies. Allergol Int 66(3):398–403. 10.1016/j.alit.2016.12.002 10.1016/j.alit.2016.12.002 28057434

[38] Radonjic-Hoesli S, Brüggen M-C, Feldmeyer L, Simon H-U, Simon D (2021) Eosinophils in skin diseases. Semin Immunopathol 43(3):393–409. 10.1007/s00281-021-00868-7 34097126 10.1007/s00281-021-00868-7 PMC8241748

[39] Karimkhani C, Silverberg JI, Dellavalle RP (2015) Defining intrinsic vs. extrinsic atopic dermatitis. Dermatol Online J 21(6):13030/qt14p8p404 26158358

[40] Zablotsky B, Black LI, Akinbami LJ (2023) Diagnosed Allergic Conditions in Children Aged 0–17 Years: United States, 2021. Jan. 10.15620/cdc:123250 36700870

[41] Laska J, Tota M, Łacwik J, Sędek Ł, Gomułka K (2024) IL-22 in atopic dermatitis. Cells 13(16):1398. 10.3390/cells13161398 39195286 10.3390/cells13161398 PMC11353104

[42] Tsoi LC et al (2020) Progression of acute-to-chronic atopic dermatitis is associated with quantitative rather than qualitative changes in cytokine responses. J Allergy Clin Immunol 145(5):1406–1415. 10.1016/j.jaci.2019.11.047 31891686 10.1016/j.jaci.2019.11.047 PMC7214216

[43] Gittler JK et al (2012) Progressive activation of Th2/Th22 cytokines and selective epidermal proteins characterizes acute and chronic atopic dermatitis. J Allergy Clin Immunol 130(6):1344–1354. 10.1016/j.jaci.2012.07.012 22951056 10.1016/j.jaci.2012.07.012 PMC3991245

[44] Langan SM, Irvine AD, Weidinger S (2020) Atopic dermatitis. Lancet 396(10247):345–360. 10.1016/S0140-6736(20)31286-1 32738956 10.1016/S0140-6736(20)31286-1

[45] Haddad E-B, Cyr SL, Arima K, McDonald RA, Levit NA, Nestle FO (2022) Current and emerging strategies to inhibit type 2 inflammation in atopic dermatitis. Dermatol Ther 12(7):1501–1533. 10.1007/s13555-022-00737-7 10.1007/s13555-022-00737-7 PMC9276864 35596901

[46] Hoyer A et al (2022) Filaggrin mutations in relation to skin barrier and atopic dermatitis in early infancy. Br J Dermatol 186(3):544–552. 10.1111/bjd.20831 34698386 10.1111/bjd.20831

[47] Palmer CNA et al (2006) Common loss-of-function variants of the epidermal barrier protein filaggrin are a major predisposing factor for atopic dermatitis. Nat Genet 38(4):441–446. 10.1038/ng1767 16550169 10.1038/ng1767

[48] Khatib CM et al (2024) Increased loss-of-function filaggrin gene mutation prevalence in atopic dermatitis patients across northern latitudes indicates genetic fitness: a systematic review and meta-analysis. Exp Dermatol 33(7):e15130. 10.1111/exd.15130 38989976 10.1111/exd.15130

[49] Aho S, Harding CR, Lee J-M, Meldrum H, Bosko CA (2012) Regulatory role for the profilaggrin N-terminal domain in epidermal homeostasis. J Invest Dermatol 132(10):2376–2385. 10.1038/jid.2012.174 22622429 10.1038/jid.2012.174

[50] Chittock J et al (2024) Association between skin barrier development and early-onset atopic dermatitis: a longitudinal birth cohort study. J Allergy Clin Immunol 153(3):732-741.e8. 10.1016/j.jaci.2023.10.017 37926123 10.1016/j.jaci.2023.10.017

[51] Miajlovic H, Fallon PG, Irvine AD, Foster TJ (2010) Effect of filaggrin breakdown products on growth of and protein expression by *Staphylococcus aureus*. J Allergy Clin Immunol 126(6):1184-1190.e3. 10.1016/j.jaci.2010.09.015 21036388 10.1016/j.jaci.2010.09.015 PMC3627960

[52] Howell MD et al (2007) Cytokine modulation of atopic dermatitis filaggrin skin expression. J Allergy Clin Immunol 120(1):150–155. 10.1016/j.jaci.2007.04.031 17512043 10.1016/j.jaci.2007.04.031 PMC2669594

[53] Hönzke S et al (2016) Influence of Th2 cytokines on the cornified envelope, tight junction proteins, and β-defensins in filaggrin-deficient skin equivalents. J Invest Dermatol 136(3):631–639. 10.1016/j.jid.2015.11.007 27015451 10.1016/j.jid.2015.11.007

[54] Barker JNWN et al (2007) Null mutations in the filaggrin gene (FLG) determine major susceptibility to early-onset atopic dermatitis that persists into adulthood. J Invest Dermatol 127(3):564–567. 10.1038/sj.jid.5700587 16990802 10.1038/sj.jid.5700587

[55] Arehart CH et al (2022) Polygenic prediction of atopic dermatitis improves with atopic training and filaggrin factors. J Allergy Clin Immunol 149(1):145–155. 10.1016/j.jaci.2021.05.034 34111454 10.1016/j.jaci.2021.05.034 PMC8973457

[56] Morar N, Cookson WOCM, Harper JI, Moffatt MF (2007) Filaggrin mutations in children with severe atopic dermatitis. J Invest Dermatol 127(7):1667–1672. 10.1038/sj.jid.5700739 17301831 10.1038/sj.jid.5700739

[57] Berdyshev E et al Lipid abnormalities in atopic skin are driven by type 2 cytokines. JCI Insight, 3, 4, p. e98006, 10.1172/jci.insight.98006 10.1172/jci.insight.98006 PMC5916244 29467325

[58] Progneaux A et al (2023) Keratinocytes activated by IL-4/IL-13 express IL-2Rγ with consequences on epidermal barrier function. Exp Dermatol 32(5):660–670. 10.1111/exd.14749 36645024 10.1111/exd.14749

[59] Gruber R et al (2011) Filaggrin genotype in ichthyosis vulgaris predicts abnormalities in epidermal structure and function. Am J Pathol 178(5):2252–2263. 10.1016/j.ajpath.2011.01.053 21514438 10.1016/j.ajpath.2011.01.053 PMC3081164

[60] Wang X, Mao D, Jia J, Zhang J (2024) Benvitimod inhibits IL-4– and IL-13–induced tight junction impairment by activating AHR/ARNT pathway and inhibiting STAT6 phosphorylation in human keratinocytes. J Invest Dermatol 144(3):509-519.e7. 10.1016/j.jid.2023.07.027 37734479 10.1016/j.jid.2023.07.027

[61] Odhiambo JA, Williams HC, Clayton TO, Robertson CF, Asher MI (2009) Global variations in prevalence of eczema symptoms in children from ISAAC Phase Three. J Allergy Clin Immunol 124(6):1251-1258.e23. 10.1016/j.jaci.2009.10.009 20004783 10.1016/j.jaci.2009.10.009

[62] Laughter MR et al (2021) The global burden of atopic dermatitis: lessons from the Global Burden of Disease Study 1990–2017*. Br J Dermatol 184(2):304–309. 10.1111/bjd.19580 33006135 10.1111/bjd.19580

[63] Chiesa Fuxench ZC et al (2019) Atopic Dermatitis in America Study: A Cross-Sectional Study Examining the Prevalence and Disease Burden of Atopic Dermatitis in the US Adult Population. J Invest Dermatol 139(3):583–590. 10.1016/j.jid.2018.08.028 30389491 10.1016/j.jid.2018.08.028

[64] Johansson EK et al (2022) Prevalence and characteristics of atopic dermatitis among young adult females and males—report from the Swedish population-based study BAMSE. J Eur Acad Dermatol Venereol 36(5):698–704. 10.1111/jdv.17929 35032357 10.1111/jdv.17929 PMC9303811

[65] Barbarot S et al (2018) Epidemiology of atopic dermatitis in adults: results from an international survey. Allergy 73(6):1284–1293. 10.1111/all.13401 29319189 10.1111/all.13401

[66] Jensen F et al (2010) Estradiol and Progesterone Regulate the Migration of Mast Cells from the Periphery to the Uterus and Induce Their Maturation and Degranulation. PLoS One 5(12):e14409. 10.1371/journal.pone.0014409 21203555 10.1371/journal.pone.0014409 PMC3008683

[67] Ng AE, Boersma MPHP and M.P.H., Products - Data Briefs - Number 460 - January 2023. Accessed: Jan. 02, 2026. [Online]. Available: https://www.cdc.gov/nchs/products/databriefs/db460.htm

[68] Fölster-Holst R, Pape M, Buss YL, Christophers E, Weichenthal M (2006) Low prevalence of the intrinsic form of atopic dermatitis among adult patients. Allergy 61(5):629–632. 10.1111/j.1398-9995.2006.01076.x 16629795 10.1111/j.1398-9995.2006.01076.x

[69] Mori T et al (2010) Comparison of skin barrier function and sensory nerve electric current perception threshold between IgE-high extrinsic and IgE-normal intrinsic types of atopic dermatitis. Br J Dermatol 162(1):83–90. 10.1111/j.1365-2133.2009.09440.x 19785593 10.1111/j.1365-2133.2009.09440.x

[70] Brenninkmeijer EEA, Spuls PI, Legierse CM, Lindeboom R, Smitt JHS, Bos JD (2008) Clinical differences between atopic and atopiform dermatitis. J Am Acad Dermatol 58(3):407–414. 10.1016/j.jaad.2007.12.002 18280337 10.1016/j.jaad.2007.12.002

[71] Kao JS et al (2001) Testosterone Perturbs Epidermal Permeability Barrier Homeostasis. J Invest Dermatol 116(3):443–451. 10.1046/j.1523-1747.2001.01281.x 10.1046/j.1523-1747.2001.01281.x 11231319

[72] Hanley K et al (1996) Hormonal basis for the gender difference in epidermal barrier formation in the fetal rat. Acceleration by estrogen and delay by testosterone. J Clin Invest 97(11): 2576–2584. 10.1172/JCI118706 10.1172/JCI118706 PMC507344 8647951

[73] Ichimasu N et al (2021) Possible involvement of type 2 cytokines in alloknesis in mouse models of menopause and dry skin. Exp Dermatol 30(12): 1745–1753. 10.1111/exd.14422 10.1111/exd.14422 34181782

[74] Hung C-F, Chen W-Y, Aljuffali IA, Lin Y-K, Shih H-C, Fang J-Y (2015) Skin aging modulates percutaneous drug absorption: the impact of ultraviolet irradiation and ovariectomy. Age 37(2):21. 10.1007/s11357-015-9757-1 25721687 10.1007/s11357-015-9757-1 PMC4342372

[75] Chen Y, Yokozeki H, Katagiri K (2017) Physiological and functional changes in the stratum corneum restored by oestrogen in an ovariectomized mice model of climacterium. Exp Dermatol 26(5):394–401. 10.1111/exd.13214 27672722 10.1111/exd.13214

[76] Narita S et al (2007) Environmental estrogens induce mast cell degranulation and enhance IgE-mediated release of allergic mediators. Environ Health Perspect. 115(1): 48–52. 10.1289/ehp.9378 10.1289/ehp.9378 PMC1797832 17366818

[77] Zaitsu M et al (2007) Estradiol activates mast cells via a non-genomic estrogen receptor-α and calcium influx. Mol Immunol 44(8): 1977–1985. 10.1016/j.molimm.2006.09.030 10.1016/j.molimm.2006.09.030 PMC2603032 17084457

[78] Mackey E, Ayyadurai S, Pohl CS, D’ Costa S, Li Y, Moeser AJ (2016) Sexual dimorphism in the mast cell transcriptome and the pathophysiological responses to immunological and psychological stress. Biol Sex Differ 7:60. 10.1186/s13293-016-0113-7 10.1186/s13293-016-0113-7 PMC5120457 27895892

[79] Cephus J-Y et al (2017) Testosterone Attenuates Group 2 Innate Lymphoid Cell-Mediated Airway Inflammation. Cell Rep 21(9): 2487–2499. 10.1016/j.celrep.2017.10.110 10.1016/j.celrep.2017.10.110 PMC5731254 29186686

[80] Blanquart E et al (2022) Targeting androgen signaling in ILC2s protects from IL-33–driven lung inflammation, independently of KLRG1. J Allergy Clin Immunol 149(1):237–251 .e12. 10.1016/j.jaci.2021.04.029 10.1016/j.jaci.2021.04.029 33964300

[81] Laffont S et al (2017) Androgen signaling negatively controls group 2 innate lymphoid cells. J Exp Med 214(6):1581–1592. 10.1084/jem.20161807 10.1084/jem.20161807 PMC5461006 28484078

[82] Carreras E et al (2010) Estrogen receptor signaling promotes dendritic cell differentiation by increasing expression of the transcription factor IRF4. Blood 115(2):238–246. 10.1182/blood-2009-08-236935 10.1182/blood-2009-08-236935 PMC2808152 19880499

[83] Paharkova-Vatchkova V, Maldonado R, Kovats S (2004) Estrogen preferentially promotes the differentiation of CD11c+ CD11bintermediate dendritic cells from bone marrow precursors1. J Immunol 172(3):1426–1436. 10.4049/jimmunol.172.3.1426 14734718 10.4049/jimmunol.172.3.1426

[84] Mao A, Paharkova-Vatchkova V, Hardy J, Miller MM, Kovats S (2005) Estrogen selectively promotes the differentiation of dendritic cells with characteristics of Langerhans cells. J Immunol Baltim Md 1950 175(8):5146–5151. 10.4049/jimmunol.175.8.5146 10.4049/jimmunol.175.8.5146 16210618

[85] Roland LT, Wise SK, Wang H, Zhang P, Mehta C, Levy JM (2021) The cost of rhinitis in the United States: a national insurance claims analysis. Int Forum Allergy Rhinol 11(5):946–948. 10.1002/alr.22748 33300670 10.1002/alr.22748 PMC8062294

[86] Bernstein JA, Bernstein JS, Makol R, Ward S (2024) Allergic rhinitis: a review. JAMA 331(10):866–877. 10.1001/jama.2024.0530 38470381 10.1001/jama.2024.0530

[87] Ozdoganoglu T, Songu M (2012) The burden of allergic rhinitis and asthma. Ther Adv Respir Dis 6(1):11–23. 10.1177/1753465811431975 22179899 10.1177/1753465811431975

[88] Bousquet P-J et al (2008) Geographical distribution of atopic rhinitis in the European Community Respiratory Health Survey I. Allergy 63(10):1301–1309. 10.1111/j.1398-9995.2008.01824.x 18782108 10.1111/j.1398-9995.2008.01824.x

[89] Savouré M, Bousquet J, Jaakkola JJK, Jaakkola MS, Jacquemin B, Nadif R (2022) Worldwide prevalence of rhinitis in adults: a review of definitions and temporal evolution. Clin Transl Allergy 12(3):e12130. 10.1002/clt2.12130 35344304 10.1002/clt2.12130 PMC8967272

[90] Licari A et al (2023) Epidemiology of allergic rhinitis in children: a systematic review and meta-analysis. J Allergy Clin Immunol Pract 11(8):2547–2556. 10.1016/j.jaip.2023.05.016 37236349 10.1016/j.jaip.2023.05.016

[91] Simoens S, Laekeman G (2009) Pharmacotherapy of allergic rhinitis: a pharmaco-economic approach. Allergy 64(1):85–95. 10.1111/j.1398-9995.2008.01909.x 19076532 10.1111/j.1398-9995.2008.01909.x

[92] Meltzer EO et al (2012) Burden of allergic rhinitis: allergies in America, Latin America, and Asia-Pacific adult surveys. Allergy Asthma Proc 33(1):S113-141. 10.2500/aap.2012.33.3603 22981425 10.2500/aap.2012.33.3603

[93] Vlastos IM, Kalentakis Z, Doulaptsi M, Karatzanis A, Prokopakis EP (2023) Multimorbidities in allergic rhinitis-current evidence from epidemiological studies, treatment trials, and molecular data. Curr Allergy Asthma Rep 23(2):133–140. 10.1007/s11882-022-01063-w 36692819 10.1007/s11882-022-01063-w

[94] Custovic A, Custovic D, Kljaić Bukvić B, Fontanella S, Haider S (2020) Atopic phenotypes and their implication in the atopic march. Expert Rev Clin Immunol 16(9):873–881. 10.1080/1744666X.2020.1816825 32856959 10.1080/1744666X.2020.1816825

[95] Del Cuvillo A et al (2017) Allergic rhinitis severity can be assessed using a visual analogue scale in mild, moderate and severe. Rhinology 55(1):34–38. 10.4193/Rhin16.025 28019644 10.4193/Rhin16.025

[96] Klimek L et al (2017) Visual analogue scales (VAS): measuring instruments for the documentation of symptoms and therapy monitoring in cases of allergic rhinitis in everyday health care: position paper of the German Society of Allergology (AeDA) and the German Society of Allergy and Clinical Immunology (DGAKI), ENT section, in collaboration with the working group on Clinical Immunology, Allergology and Environmental Medicine of the German Society of Otorhinolaryngology, Head and Neck Surgery (DGHNOKHC). Allergo J Int 26(1):16–24. 10.1007/s40629-016-0006-7 28217433 10.1007/s40629-016-0006-7 PMC5288410

[97] Bousquet J et al (2020) Allergic rhinitis. Nat Rev Dis Primer 6(1):1–17. 10.1038/s41572-020-00227-0 10.1038/s41572-020-00227-0 33273461

[98] Paller AS, Spergel JM, Mina-Osorio P, Irvine AD (2019) The atopic march and atopic multimorbidity: many trajectories, many pathways. J Allergy Clin Immunol 143(1):46–55. 10.1016/j.jaci.2018.11.006 30458183 10.1016/j.jaci.2018.11.006

[99] Belgrave DCM et al (2014) Developmental profiles of eczema, wheeze, and rhinitis: two population-based birth cohort studies. PLoS Med 11(10):e1001748. 10.1371/journal.pmed.1001748 25335105 10.1371/journal.pmed.1001748 PMC4204810

[100] Torén K, Olin AC, Hellgren J, Hermansson BA (2002) Rhinitis increase the risk for adult-onset asthma–a Swedish population-based case-control study (MAP-study). Respir Med 96(8):635–641. 10.1053/rmed.2002.1319 12195846 10.1053/rmed.2002.1319

[101] Bousquet J et al (2008) Allergic Rhinitis and its Impact on Asthma (ARIA) 2008 update (in collaboration with the World Health Organization, GA(2)LEN and AllerGen). Allergy 63:8–160. 10.1111/j.1398-9995.2007.01620.x 10.1111/j.1398-9995.2007.01620.x 18331513

[102] Huang K et al (2019) Prevalence, risk factors, and management of asthma in China: a national cross-sectional study. Lancet Lond Engl 394(10196):407–418. 10.1016/S0140-6736(19)31147-X 10.1016/S0140-6736(19)31147-X 31230828

[103] Stern J, Chen M, Fagnano M, Halterman JS (2023) Allergic rhinitis co-morbidity on asthma outcomes in city school children. J Asthma Off J Assoc Care Asthma 60(2):255–261. 10.1080/02770903.2022.2043363 10.1080/02770903.2022.2043363 PMC9653514 35195499

[104] García-Almaraz R et al (2021) Prevalence and risk factors associated with allergic rhinitis in Mexican school children: Global Asthma Network Phase I. World Allergy Organ J 14(1):100492. 10.1016/j.waojou.2020.100492 34659624 10.1016/j.waojou.2020.100492 PMC8495464

[105] Clark H et al (2019) Differential associations of allergic disease genetic variants with developmental profiles of eczema, wheeze and rhinitis. Clin Exp Allergy J Br Soc Allergy Clin Immunol 49(11):1475–1486. 10.1111/cea.13485 10.1111/cea.13485 PMC6899469 31441980

[106] Yim Y et al (2024) Sex-specific and long-term trends of asthma, allergic rhinitis, and atopic dermatitis in South Korea, 2007–2022: a nationwide representative study. Int Arch Allergy Immunol 186(2):166–183. 10.1159/000540928 39284302 10.1159/000540928 PMC11793097

[107] Huurre TM, Aro,Hillevi M, and J. J. K. and, Jaakkola (2004) Incidence and Prevalence of Asthma and Allergic Rhinitis: A Cohort Study of Finnish Adolescents. J Asthma 41(3):311–317. 10.1081/JAS-120026088 10.1081/jas-120026088 15260464

[108] Sultész M et al (2020) Prevalence of allergic rhinitis, related comorbidities and risk factors in schoolchildren. Allergy Asthma Clin Immunol Off J Can Soc Allergy Clin Immunol 16(1):98. 10.1186/s13223-020-00495-1 10.1186/s13223-020-00495-1 PMC7661153 33292450

[109] Fröhlich M et al (2017) Is there a sex-shift in prevalence of allergic rhinitis and comorbid asthma from childhood to adulthood? A meta-analysis. Clin Transl Allergy 7:44. 10.1186/s13601-017-0176-5 10.1186/s13601-017-0176-5 PMC5715620 29225773

[110] Chiu RG et al (2025) Association of Menopause and Rhinitis Among Adult Women in the United States: Findings from the All of Us Research Program. Laryngoscope 135(6):1935–1939. 10.1002/lary.32015 10.1002/lary.32015 PMC12081998 39853748

[111] Lee K, Hong Y, Choi J, Lee SH, Kim TH (2019) Life-long endogenous estrogen exposure is associated with prevalence of allergic rhinitis in postmenopausal women. Menopause 26(8):885–891. 10.1097/GME.0000000000001319 30889092 10.1097/GME.0000000000001319

[112] Liu J, Ma T, Wang X, Bai W, Wang X (2023) Associations between HT, BMI, and allergic rhinitis in perimenopausal women. Allergy Asthma Clin Immunol Off J Can Soc Allergy Clin Immunol 19(1):107. 10.1186/s13223-023-00839-7 10.1186/s13223-023-00839-7 PMC10729323 38115026

[113] Cephus J-Y et al (2021) Estrogen receptor-α signaling increases allergen-induced IL-33 release and airway inflammation. Allergy 76(1):255–268. 10.1111/all.14491 32648964 10.1111/all.14491 PMC7790897

[114] Lam K, Schleimer R, Kern RC (2015) The etiology and pathogenesis of chronic rhinosinusitis: a review of current hypotheses. Curr Allergy Asthma Rep 15(7):41. 10.1007/s11882-015-0540-2 26143392 10.1007/s11882-015-0540-2 PMC4874491

[115] Fokkens WJ et al (2012) European Position Paper on Rhinosinusitis and Nasal Polyps 2012, Rhinol Suppl 23: 3 p preceding table of contents, 1–298 22764607

[116] Dietz de Loos DAE, Hopkins C, Fokkens WJ (2013) Symptoms in chronic rhinosinusitis with and without nasal polyps. Laryngoscope 123(1):57–63. 10.1002/lary.23671 23280941 10.1002/lary.23671

[117] Delemarre T et al (2020) Type 2 inflammation in chronic rhinosinusitis without nasal polyps: another relevant endotype. J Allergy Clin Immunol 146(2):337-343.e6. 10.1016/j.jaci.2020.04.040 32417132 10.1016/j.jaci.2020.04.040

[118] Shi L-L et al (2013) Features of airway remodeling in different types of Chinese chronic rhinosinusitis are associated with inflammation patterns. Allergy 68(1):101–109. 10.1111/all.12064 23157215 10.1111/all.12064

[119] Cho SH, Kim DW, Gevaert P (2016) Chronic rhinosinusitis without nasal polyps. J Allergy Clin Immunol Pract 4(4):575–582. 10.1016/j.jaip.2016.04.015 27393771 10.1016/j.jaip.2016.04.015 PMC4939221

[120] Ference EH et al (2015) Commentary on gender differences in prevalence, treatment, and quality of life of patients with chronic rhinosinusitis. Allergy Rhinol 6(2):e82–e88. 10.2500/ar.2015.6.0120 10.2500/ar.2015.6.0120 PMC4541639 26302727

[121] Tan BK et al (2013) Incidence and associated premorbid diagnoses of patients with chronic rhinosinusitis. J Allergy Clin Immunol 131(5):1350–1360. 10.1016/j.jaci.2013.02.002 23541327 10.1016/j.jaci.2013.02.002 PMC3788631

[122] Kim DH, Han K, Kim SW (2016) Effect of chronic rhinosinusitis with or without nasal polyp on quality of life in South Korea: 5th Korea national health and nutrition examination survey Korean. Clin Exp Otorhinolaryngol 9(2):150–156. 10.21053/ceo.2015.01053 27090274 10.21053/ceo.2015.01053 PMC4881329

[123] Jurlin L et al (2019) Cluster analysis of chronic rhinosinusitis suggests gender-based differences. ORL 81(1):1–9. 10.1159/000492966 30458446 10.1159/000492966

[124] Herrera K, Parikh M, Vemula S, Hur K (2024) Effect of hormone replacement therapy on chronic rhinosinusitis management. Laryngoscope 134(9):3921–3926. 10.1002/lary.31433 38554029 10.1002/lary.31433 PMC11305951

[125] Stevens WW, Schleimer RP, Kern RC (2016) Chronic rhinosinusitis with nasal polyps. J Allergy Clin Immunol Pract 4(4):565–572. 10.1016/j.jaip.2016.04.012 27393770 10.1016/j.jaip.2016.04.012 PMC4939220

[126] Bochner BS, Stevens WW (2020) Biology and function of eosinophils in chronic rhinosinusitis with or without nasal polyps. Allergy Asthma Immunol Res 13(1):8–22. 10.4168/aair.2021.13.1.8 10.4168/aair.2021.13.1.8 PMC7680832 33191674

[127] Stevens WW et al (2015) A retrospective, cross-sectional study reveals that women with CRSwNP have more severe disease than men. Immun Inflamm Dis 3(1):14–22. 10.1002/iid3.46 25866636 10.1002/iid3.46 PMC4386911

[128] Drake-Lee AB, Lowe D, Swanston A, Grace A (1984) Clinical profile and recurrence of nasal polyps. J Laryngol Otol 98(8):783–793. 10.1017/s0022215100147462 6470574 10.1017/s0022215100147462

[129] Collins MM, Pang Y-T, Loughran S, Wilson JA (2002) Environmental risk factors and gender in nasal polyposis. Clin Otolaryngol Allied Sci 27(5):314–317. 10.1046/j.1365-2273.2002.00573.x 12383287 10.1046/j.1365-2273.2002.00573.x

[130] To T et al (2012) Global asthma prevalence in adults: findings from the cross-sectional world health survey. BMC Public Health 12:204. 10.1186/1471-2458-12-204 22429515 10.1186/1471-2458-12-204 PMC3353191

[131] Nurmagambetov T, Kuwahara R, Garbe P (2018) The economic burden of asthma in the United States, 2008–2013. Ann Am Thorac Soc 15(3):348–356. 10.1513/AnnalsATS.201703-259OC 29323930 10.1513/AnnalsATS.201703-259OC

[132] Knudsen TB, Thomsen SF, Nolte H, Backer V (2009) A population-based clinical study of allergic and non-allergic asthma. J. Asthma Off. J. Assoc. Care Asthma 46(1):91–94. 10.1080/02770900802524657 10.1080/02770900802524657 19191145

[133] Ghrairi N, Elhechmi YZ (2025) Physiopathology of allergic asthma: a comprehensive review. Scand J Immunol 101(5):e70032. 10.1111/sji.70032 40401813 10.1111/sji.70032

[134] Gauvreau GM et al (2023) Sounding the alarmins—The role of alarmin cytokines in asthma. Allergy 78(2):402–417. 10.1111/all.15609 10.1111/all.15609 PMC10108333 36463491

[135] Lund S, Walford HH, Doherty TA (2013) Type 2 innate lymphoid cells in allergic disease. Curr Immunol Rev 9(4):214–221. 10.2174/1573395510666140304235916 24876829 10.2174/1573395510666140304235916 PMC4033554

[136] Program P and T. E. P. on the D. and, Asthma M Sect. 2, Definition, Pathophysiology and Pathogenesis of Asthma, and Natural History of Asthma, in *Expert Panel Report 3: Guidelines for the Diagnosis and Management of Asthma*, National Heart, Lung, and Blood Institute (US), 2007. Accessed: Jun. 12, 2025. [Online]. Available: https://www.ncbi.nlm.nih.gov/books/NBK7223/

[137] Mega S et al (2013) Percutaneous closure of patent foramen ovale in a patient with situs viscerum inversus. J Cardiovasc Med Hagerstown Md 14(2):168–170. 10.2459/JCM.0b013e328353809d 10.2459/JCM.0b013e328353809d 22609870

[138] Fu L et al (2014) Natural Progression of Childhood Asthma Symptoms and Strong Influence of Sex and Puberty. Ann Am Thorac Soc 11(6):939–944. 10.1513/AnnalsATS.201402-084OC 10.1513/AnnalsATS.201402-084OC PMC4213994 24896645

[139] Vink NM, Postma DS, Schouten JP, Rosmalen JGM, Boezen HM (2010) Gender differences in asthma development and remission during transition through puberty: the TRacking adolescents’ individual lives survey (TRAILS) study. J Allergy Clin Immunol 126(3):498-504.e1–6. 10.1016/j.jaci.2010.06.018 20816186 10.1016/j.jaci.2010.06.018

[140] Mead J (1980) Dysanapsis in normal lungs assessed by the relationship between maximal flow, static recoil, and vital capacity. Am Rev Respir Dis 121(2):339–342. 10.1164/arrd.1980.121.2.339 7362140 10.1164/arrd.1980.121.2.339

[141] Gal-Oz ST et al (2019) ImmGen report: sexual dimorphism in the immune system transcriptome. Nat Commun 10(1):4295. 10.1038/s41467-019-12348-6 31541153 10.1038/s41467-019-12348-6 PMC6754408

[142] Yang CX et al (2019) Widespread Sexual Dimorphism in the Transcriptome of Human Airway Epithelium in Response to Smoking. Sci Rep 9(1):17600. 10.1038/s41598-019-54051-y 10.1038/s41598-019-54051-y PMC6879662 31772224

[143] Pilling D, Consalvo KM, Kirolos SA, Gomer RH (2025) Differences Between Unstimulated and Stimulated Human Male and Female Neutrophils in Protein and Phosphoprotein Profiles. Proteomics 25(7):e202400232. 10.1002/pmic.202400232 10.1002/pmic.202400232 PMC12344562 39937132

[144] Artham S, Chang C-Y, McDonnell DP (2023) Eosinophilia in cancer and its regulation by sex hormones. Trends Endocrinol Metab 34(1):5–20. 10.1016/j.tem.2022.11.002 36443206 10.1016/j.tem.2022.11.002 PMC10122120

[145] Abdullah M et al (2012) Gender effect on *in vitro* lymphocyte subset levels of healthy individuals. Cell Immunol 272(2):214–219. 10.1016/j.cellimm.2011.10.009 22078320 10.1016/j.cellimm.2011.10.009

[146] Borrelli R et al (2025) Sex-based differences in asthma: pathophysiology, hormonal influence, and genetic mechanisms. Int J Mol Sci 26(11):5288. 10.3390/ijms26115288 40508095 10.3390/ijms26115288 PMC12154264

[147] Senna G et al (2020) Sex Differences in Severe Asthma: Results From Severe Asthma Network in Italy-SANI, *Allergy Asthma Immunol. Res.*, vol. 13, no. 2, pp. 219–228, Oct. 10.4168/aair.2021.13.2.219 10.4168/aair.2021.13.2.219 PMC7840868 33474857

[148] Jenkins CR, Boulet L-P, Lavoie KL, Raherison-Semjen C, Singh D (2022) Personalized treatment of asthma: the importance of sex and gender differences. J Allergy Clin Immunol Pract 10(4):963-971.e3. 10.1016/j.jaip.2022.02.002 35150902 10.1016/j.jaip.2022.02.002

[149] Chowdhury NU, Guntur VP, Newcomb DC, Wechsler ME (2021) Sex and gender in asthma. Eur Respir Rev 30(162):210067. 10.1183/16000617.0067-2021 34789462 10.1183/16000617.0067-2021 PMC8783601

[150] Triebner K et al (2016) Menopause as a predictor of new-onset asthma: A longitudinal Northern European population study. J Allergy Clin Immunol 137(1):50–57 .e6. 10.1016/j.jaci.2015.08.019 10.1016/j.jaci.2015.08.019 26435006

[151] Zaibi H, Touil A, Fessi R, Ben Amar J, Aouina H (2020) Asthma in menopausal women: clinical and functional particularities. Tanaffos 19(3):216–222 33815542 PMC8008417

[152] DeBoer MD et al (2018) Effects of endogenous sex hormones on lung function and symptom control in adolescents with asthma. BMC Pulm Med 18(1):58. 10.1186/s12890-018-0612-x 10.1186/s12890-018-0612-x PMC5891903 29631584

[153] Wenzel SE, Robinson CB, Leonard JM, Panettieri RA (2010) Nebulized dehydroepiandrosterone-3-sulfate improves asthma control in the moderate-to-severe asthma results of a 6-week, randomized, double-blind, placebo-controlled study. Allergy Asthma Proc 31(6):461–471. 10.2500/aap.2010.31.3384 21708057 10.2500/aap.2010.31.3384

[154] Marozkina N et al (2019) Dehydroepiandrosterone supplementation may benefit women with asthma who have low androgen levels: a pilot study. Pulm Ther 5(2):213–220. 10.1007/s41030-019-00101-9 32026412 10.1007/s41030-019-00101-9 PMC6967310

[155] Pate CA (2021) Asthma surveillance — United States, 2006–2018. MMWR Surveill Summ. 10.15585/mmwr.ss7005a1 34529643 10.15585/mmwr.ss7005a1 PMC8480992

[156] Gupta RS et al (2019) Prevalence and severity of food allergies among US adults. JAMA Netw Open 2(1):e185630. 10.1001/jamanetworkopen.2018.5630 30646188 10.1001/jamanetworkopen.2018.5630 PMC6324316

[157] Wood RA et al (2024) Omalizumab for the treatment of multiple food allergies. N Engl J Med 390(10):889–899. 10.1056/NEJMoa2312382 38407394 10.1056/NEJMoa2312382 PMC11193494

[158] Hill DA, Spergel JM (2018) The atopic march: critical evidence and clinical relevance. Ann Allergy Asthma Immunol Off Publ Am Coll Allergy Asthma Immunol 120(2):131–137. 10.1016/j.anai.2017.10.037 10.1016/j.anai.2017.10.037 PMC5806141 29413336

[159] Giovannini M, Skypala IJ, Caubet JC, Toit GD, Nowak-Wegrzyn A (2024) Diagnosis and management of pollen food allergy syndrome to nuts. J Allergy Clin Immunol Pract 12(3):599–604. 10.1016/j.jaip.2024.01.025 38280450 10.1016/j.jaip.2024.01.025

[160] Lieberman J, Muraro A, Blaiss M (2024) How to diagnose IgE-mediated food allergy. Arch Dis Child-Educ Pract 109(5):247–251. 10.1136/archdischild-2023-325938 10.1136/archdischild-2023-325938 PMC11503118 38453428

[161] Sicherer SH, Muñoz-Furlong A, Godbold JH, Sampson HA (2010) US prevalence of self-reported peanut, tree nut, and sesame allergy: 11-year follow-up. J Allergy Clin Immunol 125(6):1322–1326. 10.1016/j.jaci.2010.03.029 20462634 10.1016/j.jaci.2010.03.029

[162] Warren C, Lei D, Sicherer S, Schleimer R, Gupta R (2021) Prevalence and characteristics of peanut allergy in US adults. J Allergy Clin Immunol 147(6):2263-2270.e5. 10.1016/j.jaci.2020.11.046 33579526 10.1016/j.jaci.2020.11.046 PMC12341317

[163] Suaini NHA, Siah KTH, Tham EH (2021) Role of the gut-skin axis in IgE-mediated food allergy and atopic diseases. Curr Opin Gastroenterol 37(6):557–564. 10.1097/MOG.0000000000000780 34411036 10.1097/MOG.0000000000000780

[164] Haidar L et al (2025) Beyond the skin: exploring the gut–skin axis in chronic spontaneous urticaria and other inflammatory skin diseases. Biomedicines. 10.3390/biomedicines13082014 40868265 10.3390/biomedicines13082014 PMC12383297

[165] Jang J-H et al (2024) Chronic gut inflammation and dysbiosis in IBS: unraveling their contribution to atopic dermatitis progression. Int J Mol Sci. 10.3390/ijms25052753 38473999 10.3390/ijms25052753 PMC10931664

[166] Martin PE et al (2015) Which infants with eczema are at risk of food allergy? Results from a population-based cohort. Clin Exp Allergy 45(1):255–264. 10.1111/cea.12406 25210971 10.1111/cea.12406

[167] Thye A-K et al (2022) Gut–skin axis: unravelling the connection between the gut microbiome and psoriasis. Biomedicines. 10.3390/biomedicines10051037 35625774 10.3390/biomedicines10051037 PMC9138548

[168] Islam SA, Luster AD (2012) T cell homing to epithelial barriers in allergic disease. Nat Med 18(5):705–715. 10.1038/nm.2760 22561834 10.1038/nm.2760 PMC3863331

[169] Hou B, Shao H, Yuan D, Tham EH (2025) Skin and gut microbiome in atopic dermatitis: Mechanisms and therapeutic opportunities. Pediatr Allergy Immunol 36(12):e70265. 10.1111/pai.70265 41388767 10.1111/pai.70265 PMC12701371

[170] Clark RA (2010) Skin-resident T cells: the ups and downs of on site immunity. J Invest Dermatol 130(2):362–370. 10.1038/jid.2009.247 19675575 10.1038/jid.2009.247 PMC2922675

[171] van Splunter M et al (2020) Mechanisms underlying the skin-gut cross talk in the development of IgE-mediated food allergy. Nutrients. 10.3390/nu12123830 33333859 10.3390/nu12123830 PMC7765270

[172] Leyva-Castillo J-M et al (2019) Mechanical skin injury promotes food anaphylaxis by driving intestinal mast cell expansion. Immunity 50(5):1262-1275.e4. 10.1016/j.immuni.2019.03.023 31027995 10.1016/j.immuni.2019.03.023 PMC6531322

[173] Xue Y, Zhang L, Chen Y, Wang H, Xie J (2023) Gut microbiota and atopic dermatitis: a two-sample Mendelian randomization study. Front Med. 10.3389/fmed.2023.1174331 37425302 10.3389/fmed.2023.1174331 PMC10323683

[174] Vininski MS, Rajput S, Hobbs NJ, Dolence JJ (2022) Understanding sex differences in the allergic immune response to food. AIMS Allergy Immunol 6(3):90–105. 10.3934/allergy.2022009 38314333 10.3934/allergy.2022009 PMC10836331

[175] Kelly C, Gangur V (2009) Sex disparity in food allergy: evidence from the PubMed database. J Allergy 2009(1):159845. 10.1155/2009/159845 10.1155/2009/159845 PMC2957586 20975795

[176] Wang J et al (2021) Gender differences in food allergy depend on the PPAR γ/NF-κB in the intestines of mice. Life Sci 278:119606. 10.1016/j.lfs.2021.119606 33974930 10.1016/j.lfs.2021.119606

[177] Hox V, Desai A, Bandara G, Gilfillan AM, Metcalfe DD, Olivera A (2015) Estrogen increases the severity of anaphylaxis in female mice through enhanced endothelial nitric oxide synthase expression and nitric oxide production. J Allergy Clin Immunol 135(3):729-736.e5. 10.1016/j.jaci.2014.11.003 25553642 10.1016/j.jaci.2014.11.003 PMC5586107

[178] Vliagoftis H et al (1992) Estradiol augments while tamoxifen inhibits rat mast cell secretion. Int Arch Allergy Immunol 98(4):398–409. 10.1159/000236217 1384869 10.1159/000236217

[179] Muñoz-Cruz S, Mendoza-Rodríguez Y, Nava-Castro KE, Yepez-Mulia L, Morales-Montor J (2015) Gender-related effects of sex steroids on histamine release and FcεRI expression in rat peritoneal mast cells. J Immunol Res 2015:351829. 10.1155/2015/351829 25973435 10.1155/2015/351829 PMC4417946

[180] Kim MS, Chae HJ, Shin TY, Kim HM, Kim HR (2001) Estrogen regulates cytokine release in human mast cells. Immunopharmacol Immunotoxicol 23(4):495–504. 10.1081/iph-100108596 11792009 10.1081/iph-100108596

[181] Poonawalla T, Kelly B (2009) Urticaria : a review. Am J Clin Dermatol 10(1):9–21. 10.2165/0128071-200910010-00002 19170406 10.2165/0128071-200910010-00002

[182] Cherrez Ojeda I et al (2018) Etiology of chronic urticaria: the Ecuadorian experience. World Allergy Organ J 11:1. 10.1186/s40413-017-0181-0 29308115 10.1186/s40413-017-0181-0 PMC5753451

[183] Dabija D, Tadi P, Danosos GN, Chronic, Urticaria in *StatPearls*, Treasure Island (FL): StatPearls Publishing, 2025. Accessed: Jun. 12, 2025. [Online]. Available: http://www.ncbi.nlm.nih.gov/books/NBK555910/

[184] Kolkhir P, Giménez-Arnau AM, Kulthanan K, Peter J, Metz M, Maurer M (2022) Urticaria. Nat Rev Dis Primer 8(1):61. 10.1038/s41572-022-00389-z 10.1038/s41572-022-00389-z 36109590

[185] Geissbühler Y et al (2025) Incidence and Prevalence of Chronic Spontaneous Urticaria Among Adult and Pediatric Populations in the United States. Adv Ther 42(6):2808–2820. 10.1007/s12325-025-03172-0 40238059 10.1007/s12325-025-03172-0 PMC12085360

[186] Xiang Y-K et al (2023) Most Patients With Autoimmune Chronic Spontaneous Urticaria Also Have Autoallergic Urticaria, but Not ViceVersa, *J. Allergy Clin. Immunol. Pract.*, vol. 11, no. 8, pp. 2417–2425.e1, Aug. 10.1016/j.jaip.2023.02.006 10.1016/j.jaip.2023.02.006 36805105

[187] Grattan CEH, Sabroe RA, Greaves MW (2002) Chronic urticaria. J Am Acad Dermatol 46(5):645–660. 10.1067/mjd.2002.122759 12004303 10.1067/mjd.2002.122759

[188] Fusari A, Colangelo C, Bonifazi F, Antonicelli L (2005) The autologous serum skin test in the follow-up of patients with chronic urticaria. Allergy 60(2):256–258. 10.1111/j.1398-9995.2005.00673.x 15647050 10.1111/j.1398-9995.2005.00673.x

[189] Kocatürk E, Kavala M, Kural E, Sarıgul S, Zındancı I (2011) Autologous serum skin test vs autologous plasma skin test in patients with chronic urticaria: evaluation of reproducibility, sensitivity and specificity and relationship with disease activity, quality of life and anti-thyroid antibodies. Eur J Dermatol EJD 21(3):339–343. 10.1684/ejd.2011.1294 21697031 10.1684/ejd.2011.1294

[190] Larenas-Linnemann D (2023) Biomarkers of autoimmune chronic spontaneous urticaria. Curr Allergy Asthma Rep 23(12):655–664. 10.1007/s11882-023-01117-7 38064133 10.1007/s11882-023-01117-7

[191] Muñoz M, Kiefer LA, Pereira MP, Bizjak M, Maurer M (2024) New insights into chronic inducible urticaria. Curr Allergy Asthma Rep 24(8):457–469. 10.1007/s11882-024-01160-y 39028396 10.1007/s11882-024-01160-y PMC11297124

[192] Fricke J et al (2020) Prevalence of chronic urticaria in children and adults across the globe: systematic review with meta-analysis. Allergy 75(2):423–432. 10.1111/all.14037 31494963 10.1111/all.14037

[193] Cassano N, Colombo D, Bellia G, Zagni E, Vena GA (2016) Gender-related differences in chronic urticaria. G. Ital. Dermatol. E Venereol. Organo Uff. Soc. Ital. Dermatol. E Sifilogr. 151(5):544–552 26091277

[194] Kocatürk E et al (2025) Sex matters in CSU: Women face greater burden and poorer urticaria control, especially in midlife-CURE insights. J Eur Acad Dermatol Venereol JEADV 40(1): 67–78. 10.1111/jdv.70027 10.1111/jdv.70027 PMC12723574 40965122

[195] Erol K, Ertaş ŞK, Ertaş R (2021) Fatigue is common and predicted by female gender and sleep disturbance in patients with chronic spontaneous urticaria. J Allergy Clin Immunol Pract 9(1):469–476. 10.1016/j.jaip.2020.08.020 32858240 10.1016/j.jaip.2020.08.020

[196] Sánchez-Díaz M, Salazar-Nievas MC, Molina-Leyva A, Arias-Santiago S (2023) Risk factors of quality-of-life and sexual function impairment in chronic spontaneous urticaria patients: cross-sectional study. Dermatol Basel Switz 239(4):601–608. 10.1159/000530518 10.1159/000530518 37019095

[197] Gregoriou S et al (2009) Etiologic Aspects and Prognostic Factors of Patients with Chronic Urticaria: Nonrandomized, Prospective, Descriptive Study. J Cutan Med Surg 13(4): 198–203. 10.2310/7750.2008.08035 10.2310/7750.2008.08035 19706227

[198] Sirufo MM, Bassino EM, De Pietro F, Ginaldi L, De Martinis M (2021) Sex differences in the efficacy of omalizumab in the treatment of chronic spontaneous urticaria. Int J Immunopathol Pharmacol 35:20587384211065870. 10.1177/20587384211065870 35170369 10.1177/20587384211065870 PMC8855371

[199] Limphoka P, Jiamton S, Chularojanamontri L, Kulthanan K, Tuchinda P (2025) Recurrent chronic spontaneous urticaria in a tropical country: clinical characteristics and associated factors. Asian Pac J Allergy Immunol 43(3):513–520. 10.12932/AP-261124-1984 40321140 10.12932/AP-261124-1984

[200] Grieco T et al (2020) Potential clinical and serological predictors of chronic spontaneous urticaria relapse in patients under omalizumab treatment. Immunotherapy 12(16):1173–1181. 10.2217/imt-2020-0088 32892673 10.2217/imt-2020-0088

[201] Ornek SA, Alkilinc AS, Kızıltac U, Kızıltac K, Kocaturk E (2023) Effect of puberty, menstruation, pregnancy, lactation, and menopause on chronic urticaria activity. J Cutan Med Surg 27(5):466–471. 10.1177/12034754231191472 37537974 10.1177/12034754231191472

[202] Kasperska-Zajac A, Brzoza Z, Rogala B (2008) Sex hormones and urticaria. J Dermatol Sci 52(2):79–86. 10.1016/j.jdermsci.2008.04.002 18485675 10.1016/j.jdermsci.2008.04.002

[203] Roberts LJ (1979) Recurrent syncope due to systemic mastocytosis, Hypertens. Dallas Tex 6(2)Pt 1, pp. 285–294, 1984, vol. 6, no. 2 Pt 1, pp. 285–294, 1984 6202635

[204] Broesby-Olsen S et al (2016) Risk of solid cancer, cardiovascular disease, anaphylaxis, osteoporosis and fractures in patients with systemic mastocytosis: a nationwide population‐based study. Am J Hematol 91(11):1069–1075. 10.1002/ajh.24490 27428296 10.1002/ajh.24490

[205] Bergström A, Hägglund H, Berglund A, Nilsson G, Lambe M (2024) Epidemiology of mastocytosis: a population-based study (Sweden). Acta Oncol 63:44–50. 10.2340/1651-226X.2024.31406 38380845 10.2340/1651-226X.2024.31406 PMC11332469

[206] Zanotti R et al (2021) A multidisciplinary diagnostic approach reveals a higher prevalence of indolent systemic mastocytosis: 15-years’ experience of the GISM network. Cancers 13(24):6380. 10.3390/cancers13246380 34944999 10.3390/cancers13246380 PMC8699786

[207] Kluin-Nelemans HC et al (2021) Cytogenetic and molecular aberrations and worse outcome for male patients in systemic mastocytosis. Theranostics 11(1):292–303. 10.7150/thno.51872 33391475 10.7150/thno.51872 PMC7681091

[208] Häder T, Molderings GJ, Klawonn F, Conrad R, Mücke M, Sellin J (2023) Cluster-analytic identification of clinically meaningful subtypes in MCAS: the relevance of heat and cold. Dig Dis Sci 68(8):3400–3412. 10.1007/s10620-023-07921-5 37029308 10.1007/s10620-023-07921-5 PMC10352424

[209] Puxkandl V, Aigner S, Hoetzenecker W, Altrichter S (2024) Hereditary alpha tryptasemia: elevated tryptase, female sex, thyroid disorders, and anaphylaxis. Front Allergy 5:1461359. 10.3389/falgy.2024.1461359 39600380 10.3389/falgy.2024.1461359 PMC11588693

[210] Ungerstedt J, Ljung C, Klimkowska M, Gülen T (2022) Clinical outcomes of adults with systemic mastocytosis: a 15-year multidisciplinary experience. Cancers 14(16):3942. 10.3390/cancers14163942 36010937 10.3390/cancers14163942 PMC9405903

[211] Novak P, Giannetti MP, Weller E, Hamilton MJ, Castells M (2022) Mast cell disorders are associated with decreased cerebral blood flow and small fiber neuropathy. Ann Allergy Asthma Immunol 128(3):299-306.e1. 10.1016/j.anai.2021.10.006 34648976 10.1016/j.anai.2021.10.006

[212] Lim K-H et al (2009) Systemic mastocytosis in 342 consecutive adults: survival studies and prognostic factors. Blood 113(23):5727–5736. 10.1182/blood-2009-02-205237 19363219 10.1182/blood-2009-02-205237

[213] Robey RC et al (2020) Hereditary Alpha-Tryptasemia: UK Prevalence and Variability in Disease Expression. J Allergy Clin Immunol Pract 8(10):3549–3556. 10.1016/j.jaip.2020.05.057 10.1016/j.jaip.2020.05.057 32553831

[214] Hamilton MJ et al (2021) Distinct small intestine mast cell histologic changes in patients with hereditary alpha-tryptasemia and mast cell activation syndrome. Am J Surg Pathol 45(7):997. 10.1097/PAS.0000000000001676 33481382 10.1097/PAS.0000000000001676 PMC8192345

[215] Cohen SS et al (2014) Epidemiology of systemic mastocytosis in Denmark. Br J Haematol 166(4):521–528. 10.1111/bjh.12916 24761987 10.1111/bjh.12916

[216] Molderings GJ, Zienkiewicz T, Homann J, Menzen M, Afrin LB (2017) Risk of solid cancer in patients with mast cell activation syndrome: results from Germany and USA. F1000Res 6:1889. 10.12688/f1000research.12730.1 29225779 10.12688/f1000research.12730.1 PMC5710302

[217] Giannetti MP, Weller E, Bormans C, Novak P, Hamilton MJ, Castells M (2021) Hereditary alpha-tryptasemia in 101 patients with mast cell activation–related symptomatology including anaphylaxis. Ann Allergy Asthma Immunol 126(6):655–660. 10.1016/j.anai.2021.01.016 33465452 10.1016/j.anai.2021.01.016

[218] Chollet MB, Akin C (2022) Hereditary alpha tryptasemia is not associated with specific clinical phenotypes. J Allergy Clin Immunol 149(2):728-735.e2. 10.1016/j.jaci.2021.06.017 34174297 10.1016/j.jaci.2021.06.017

[219] Molderings GJ, Haenisch B, Bogdanow M, Fimmers R, Nöthen MM (2013) Familial occurrence of systemic mast cell activation disease. PLoS One 8(9):e76241. 10.1371/journal.pone.0076241 24098785 10.1371/journal.pone.0076241 PMC3787002

[220] Afrin LB, Self S, Menk J, Lazarchick J (2017) Characterization of mast cell activation syndrome. Am J Med Sci 353(3):207–215. 10.1016/j.amjms.2016.12.013 28262205 10.1016/j.amjms.2016.12.013 PMC5341697

[221] Lyons JJ et al (2014) Mendelian inheritance of elevated serum tryptase associated with atopy and connective tissue abnormalities. J Allergy Clin Immunol 133(5):1471–1474. 10.1016/j.jaci.2013.11.039 10.1016/j.jaci.2013.11.039 PMC4016972 24472624

[222] Sabato V et al (2014) Familial hypertryptasemia with associated mast cell activation syndrome. J Allergy Clin Immunol 134(6):1448–1450e. 10.1016/j.jaci.2014.06.007 10.1016/j.jaci.2014.06.007 25086867

[223] Lyons JJ et al (2016) Elevated basal serum tryptase identifies a multisystem disorder associated with increased TPSAB1 copy number. Nat Genet 48(12):1564–1569. 10.1038/ng.3696 10.1038/ng.3696 PMC5397297 27749843

[224] Luskin KT, White AA, Lyons JJ (2021) The Genetic Basis and Clinical Impact of Hereditary Alpha-Tryptasemia. J Allergy Clin Immunol Pract 9(6):2235–2242. 10.1016/j.jaip.2021.03.005 33744473 10.1016/j.jaip.2021.03.005

[225] Glover SC et al (2021) Clinical relevance of inherited genetic differences in human tryptases. Ann Allergy Asthma Immunol 127(6):638–647. 10.1016/j.anai.2021.08.009 34400315 10.1016/j.anai.2021.08.009 PMC9413800

[226] Polivka L et al (2023) Pathophysiologic implications of elevated prevalence of hereditary alpha-tryptasemia in all mastocytosis subtypes. J Allergy Clin Immunol 153(1):349–353. 10.1016/j.jaci.2023.08.015.e4 37633651

[227] Soto D, Malmsten C, Blount JL, Muilenburg DJ, Caughey GH (2002) Genetic deficiency of human mast cell alpha-tryptase. Clin Exp Allergy J Br Soc Allergy Clin Immunol 32(7):1000–1006. 10.1046/j.1365-2222.2002.01416.x 10.1046/j.1365-2222.2002.01416.x 12100045

[228] Le QT et al (2019) Impact of naturally forming human α/β-tryptase heterotetramers in the pathogenesis of hereditary α-tryptasemia. J Exp Med 216(10): 2348–2361. 10.1084/jem.20190701 10.1084/jem.20190701 PMC6780998 31337736

[229] Lyons JJ et al (2021) Heritable risk for severe anaphylaxis associated with increased α-tryptase-encoding germline copy number at TPSAB1. J Allergy Clin Immunol 147(2):622–632. 10.1016/j.jaci.2020.06.035 10.1016/j.jaci.2020.06.035 32717252

[230] Vazquez M et al (2022) Hereditary alpha-tryptasemia modifies clinical phenotypes among individuals with congenital hypermobility disorders. HGG Adv 3(2): 100094. 10.1016/j.xhgg.2022.100094 10.1016/j.xhgg.2022.100094 PMC8917312 35287299

[231] Castells M et al (2024) Mast cell activation syndrome: Current understanding and research needs. J Allergy Clin Immunol 154(2):255–263. 10.1016/j.jaci.2024.05.025 10.1016/j.jaci.2024.05.025 PMC11881543 38851398

[232] Hamilton MJ, Hornick JL, Akin C, Castells MC, Greenberger NJ (2011) Mast cell activation syndrome: a newly recognized disorder with systemic clinical manifestations. J Allergy Clin Immunol 128(1):147-152.e2. 10.1016/j.jaci.2011.04.037 21621255 10.1016/j.jaci.2011.04.037

[233] Bonadonna P, Nalin F, Olivieri F (2022) Hereditary alpha-tryptasemia. Curr Opin Allergy Clin Immunol 22(5):277–282. 10.1097/ACI.0000000000000849 35942852 10.1097/ACI.0000000000000849

[234] Sonneck K et al (2007) Diagnostic and subdiagnostic accumulation of mast cells in the bone marrow of patients with anaphylaxis: monoclonal mast cell activation syndrome. Int Arch Allergy Immunol 142(2):158–164. 10.1159/000096442 17057414 10.1159/000096442

[235] Roberts LJ, Oates JA (1991) Biochemical diagnosis of systemic mast cell disorders. J Invest Dermatol 96(3):S19–S25. 10.1111/1523-1747.ep12468945 2002247

[236] Valent P et al (2012) Definitions, criteria and global classification of mast cell disorders with special reference to mast cell activation syndromes: a consensus proposal. Int Arch Allergy Immunol 157(3):215–225. 10.1159/000328760 22041891 10.1159/000328760 PMC3224511

[237] Weiler CR(2019) AAAAI Mast Cell Disorders Committee Work Group Report : Mast cell activation syndrome (MCAS) diagnosis and management. J Allergy Clin Immunol 144(4): 883–896. 10.1016/j.jaci.2019.08.023 10.1016/j.jaci.2019.08.023 31476322

[238] Gulen T (2024) Using the right criteria for MCAS. Curr Allergy Asthma Rep 24(2):39–51. 10.1007/s11882-024-01126-0 38243020 10.1007/s11882-024-01126-0 PMC10866766

[239] Afrin LB et al (2020) Diagnosis of mast cell activation syndrome: a global ‘consensus-2,.’ Diagnosis 8(2):137–152. 10.1515/dx-2020-0005 32324159 10.1515/dx-2020-0005

[240] Butterfield JH, Taylor A (2025) Acute/baseline ratios of all 3 MC mediator metabolites can enhance diagnosis and management of mast cell activation syndrome. J Allergy Clin Immunol Glob 4(2):100399. 10.1016/j.jacig.2024.100399 39906893 10.1016/j.jacig.2024.100399 PMC11791225

[241] Buttgereit T, Gu S, Carneiro-Leão L, Gutsche A, Maurer M, Siebenhaar F (2022) Idiopathic mast cell activation syndrome is more often suspected than diagnosed—a prospective real‐life study. Allergy 77(9):2794–2802. 10.1111/all.15304 35364617 10.1111/all.15304

[242] Solomon BD, Khatri P (2025) Clustering of clinical symptoms using large language models reveals low diagnostic specificity of proposed alternatives to consensus mast cell activation syndrome criteria. J Allergy Clin Immunol 155(1):213-218.e4. 10.1016/j.jaci.2024.09.006 39278360 10.1016/j.jaci.2024.09.006 PMC11700772

[243] Simpson G (2016) Menstrual anaphylactoid reactions and presumed systemic mast cell activation syndrome. Intern Med J 46(7):858–859. 10.1111/imj.13077 27405897 10.1111/imj.13077

[244] Serrano Villar S et al (2009) A 38 year old woman with hypotensive shock at the onset of menstruation: case outcome. BMJ 338:b247. 10.1136/bmj.b247 19273521 10.1136/bmj.b247

[245] Mackey E et al (2020) Perinatal androgens organize sex differences in mast cells and attenuate anaphylaxis severity into adulthood. Proc Natl Acad Sci 117(38):23751–23761. 10.1073/pnas.1915075117 32917815 10.1073/pnas.1915075117 PMC7519313

[246] Farley M, Estrada-Mendizabal RJ, Gansert EA, Voelker D, Marks LA, Gonzalez-Estrada A (2025) Prevalence of mast cell activation disorders and hereditary alpha tryptasemia among patients with postural orthostatic tachycardia syndrome and Ehlers-Danlos syndrome: a systematic review. Ann Allergy Asthma Immunol S1081120625001589. 10.1016/j.anai.2025.03.022 10.1016/j.anai.2025.03.022 40185471

[247] Vernino S et al (2021) Postural orthostatic tachycardia syndrome (POTS): State of the science and clinical care from a 2019 National Institutes of Health Expert Consensus Meeting - Part 1. Auton Neurosci 235:102828. 10.1016/j.autneu.2021.102828 10.1016/j.autneu.2021.102828 PMC8455420 34144933

[248] Boris JR, Shadiack EC, McCormick EM, MacMullen L, George-Sankoh I, Falk MJ (2024) Long‐term POTS outcomes survey: diagnosis, therapy, and clinical outcomes. J Am Heart Assoc 13(14):e033485. 10.1161/JAHA.123.033485 38958137 10.1161/JAHA.123.033485 PMC11292765

[249] Demmler JC, Atkinson MD, Reinhold EJ, Choy E, Lyons RA, Brophy ST (2019) Diagnosed prevalence of Ehlers-Danlos syndrome and hypermobility spectrum disorder in Wales, UK: a national electronic cohort study and case-control comparison. BMJ Open 9(11):e031365. 10.1136/bmjopen-2019-031365 31685485 10.1136/bmjopen-2019-031365 PMC6858200

[250] Hill DA, Grundmeier RW, Ramos M, Spergel JM (2018) Eosinophilic esophagitis is a late manifestation of the allergic march. J Allergy Clin Immunol Pract 6(5):1528–1533. 10.1016/j.jaip.2018.05.010 29954692 10.1016/j.jaip.2018.05.010 PMC6131029

[251] O’Shea KM et al (2018) Pathophysiology of eosinophilic esophagitis. Gastroenterology 154(2):333–345. 10.1053/j.gastro.2017.06.065 28757265 10.1053/j.gastro.2017.06.065 PMC5787048

[252] Davis BP, Rothenberg ME (2016) Mechanisms of disease of eosinophilic esophagitis. Annu Rev Pathol Mech Dis 11(1):365–393. 10.1146/annurev-pathol-012615-044241 10.1146/annurev-pathol-012615-044241 PMC4918086 26925500

[253] Furuta GT, Katzka DA (2015) Eosinophilic esophagitis. N Engl J Med 373(17):1640–1648. 10.1056/NEJMra1502863 26488694 10.1056/NEJMra1502863 PMC4905697

[254] Mansoor E, Cooper GS (2016) The 2010–2015 prevalence of eosinophilic esophagitis in the USA: a population-based study. Dig Dis Sci 61(10):2928–2934. 10.1007/s10620-016-4204-4 27250980 10.1007/s10620-016-4204-4 PMC5021560

[255] Alexander ES et al (2014) Twin and family studies reveal strong environmental and weaker genetic cues explaining heritability of eosinophilic esophagitis. J Allergy Clin Immunol 134(5):1084–1092. 10.1016/j.jaci.2014.07.021 10.1016/j.jaci.2014.07.021 PMC4253562 25258143

[256] Dellon ES, Jensen ET, Martin CF, Shaheen NJ, Kappelman MD (2014) Prevalence of eosinophilic esophagitis in the United States. Clin Gastroenterol Hepatol 12(4):589-596.e1. 10.1016/j.cgh.2013.09.008 24035773 10.1016/j.cgh.2013.09.008 PMC3952040

[257] Blanchard C (2006) Eotaxin-3 and a uniquely conserved gene-expression profile in eosinophilic esophagitis. J Clin Invest 116(2):536–547. 10.1172/JCI26679 16453027 10.1172/JCI26679 PMC1359059

[258] Sherrill JD et al (2014) Desmoglein-1 regulates esophageal epithelial barrier function and immune responses in eosinophilic esophagitis. Mucosal Immunol 7(3):718–729. 10.1038/mi.2013.90 24220297 10.1038/mi.2013.90 PMC3999291

[259] Litosh VA, Rochman M, Rymer JK, Porollo A, Kottyan LC, Rothenberg ME (2017) Calpain-14 and its association with eosinophilic esophagitis. J Allergy Clin Immunol 139(6):1762-1771.e7. 10.1016/j.jaci.2016.09.027 28131390 10.1016/j.jaci.2016.09.027 PMC5461191

[260] Rothenberg ME (2015) Molecular, genetic, and cellular bases for treating eosinophilic esophagitis. Gastroenterology 148(6):1143–1157. 10.1053/j.gastro.2015.02.002 25666870 10.1053/j.gastro.2015.02.002 PMC4409569

[261] Azouz NP et al (2018) The antiprotease SPINK7 serves as an inhibitory checkpoint for esophageal epithelial inflammatory responses. Sci Transl Med 10(444):eaap9736. 10.1126/scitranslmed.aap9736 29875205 10.1126/scitranslmed.aap9736 PMC6065103

[262] Wu L et al (2018) Filaggrin and tight junction proteins are crucial for IL-13-mediated esophageal barrier dysfunction. Am J Physiol -Gastrointest Liver Physiol 315(3):G341–G350. 10.1152/ajpgi.00404.2017 10.1152/ajpgi.00404.2017 29746170

[263] Biedermann L, Straumann A (2023) Mechanisms and clinical management of eosinophilic oesophagitis: an overview. Nat Rev Gastroenterol Hepatol 20(2):101–119. 10.1038/s41575-022-00691-x 36253463 10.1038/s41575-022-00691-x

[264] Vinit C et al (2019) Eosinophilic esophagitis: pathophysiology, diagnosis, and management. Arch Pediatr 26(3):182–190. 10.1016/j.arcped.2019.02.005 30827775 10.1016/j.arcped.2019.02.005

[265] Zhernov YV et al (2021) Molecular Mechanisms of Eosinophilic Esophagitis. Int J Mol Sci 22 24, Art. 24. 10.3390/ijms222413183 10.3390/ijms222413183 PMC8703627 34947981

[266] Lyles J, Rothenberg M (2019) Role of genetics, environment, and their interactions in the pathogenesis of eosinophilic esophagitis. Curr Opin Immunol 60:46–53. 10.1016/j.coi.2019.04.004 31132551 10.1016/j.coi.2019.04.004 PMC6800613

[267] Abonia JP et al (2010) Involvement of mast cells in eosinophilic esophagitis. J Allergy Clin Immunol 126(1): 140–149. 10.1016/j.jaci.2010.04.009 10.1016/j.jaci.2010.04.009 PMC2902643 20538331

[268] Noti M et al (2013) Thymic stromal lymphopoietin–elicited basophil responses promote eosinophilic esophagitis. Nat Med 19(8): 1005–1013. 10.1038/nm.3281 10.1038/nm.3281 PMC3951204 23872715

[269] Mona R, Hruz P (2025) Epidemiology of eosinophilic esophagitis: really a novel and evolving disease? Inflamm Intest Dis 10(1):34–40. 10.1159/000543022 39834520 10.1159/000543022 PMC11745509

[270] Sperry SL, Woosley JT, Shaheen NJ, Dellon ES (2012) Influence of race and gender on the presentation of eosinophilic esophagitis. Am J Gastroenterol 107(2):215–221. 10.1038/ajg.2011.342 21971538 10.1038/ajg.2011.342 PMC4584147

[271] Schreiner P et al (2022) Sex impacts disease activity but not symptoms or quality of life in adults with eosinophilic esophagitis. Clin Gastroenterol Hepatol 20(8):1729-1738.e1. 10.1016/j.cgh.2021.11.009 34798333 10.1016/j.cgh.2021.11.009

[272] Lynch KL et al (2016) Gender is a determinative factor in the initial clinical presentation of eosinophilic esophagitis. Dis Esophagus 29(2):174–178. 10.1111/dote.12307 25626069 10.1111/dote.12307

[273] Moawad FJ et al (2016) Effects of race and sex on features of eosinophilic esophagitis. Clin Gastroenterol Hepatol 14(1):23–30. 10.1016/j.cgh.2015.08.034 26343181 10.1016/j.cgh.2015.08.034

[274] Gonsalves N, Berdnikovs S, Schroeder H, Zalewski A, Bryce PJ (2017) Gender-specific differences in the molecular signatures of adult eosinophilic oesophagitis. Clin Exp Allergy J Br Soc Allergy Clin Immunol 47(7):969–971. 10.1111/cea.12960 10.1111/cea.12960 PMC5862544 28580626

[275] Russo RC, Quesniaux VFJ, Ryffel B (2023) Homeostatic chemokines as putative therapeutic targets in idiopathic pulmonary fibrosis. Trends Immunol 44(12):1014–1030. 10.1016/j.it.2023.10.003 37951789 10.1016/j.it.2023.10.003

[276] Feltenmark S et al (2008) Eoxins are proinflammatory arachidonic acid metabolites produced via the 15-lipoxygenase-1 pathway in human eosinophils and mast cells. Proc Natl Acad Sci 105(2): 680–685. 10.1073/pnas.0710127105 10.1073/pnas.0710127105 PMC2206596 18184802

[277] Schneider J, Scheibling C, Segall D, Sambursky R, Ohsfeldt R, Lovejoy L (2014) Epidemiology and economic burden of conjunctivitis: a managed care perspective. J Manag Care Med 17(1):78–83

[278] Singh K, Axelrod S, Bielory L (2010) The epidemiology of ocular and nasal allergy in the United States, 1988–1994. J Allergy Clin Immunol 126(4):778-783.e6. 10.1016/j.jaci.2010.06.050 20920769 10.1016/j.jaci.2010.06.050

[279] Miyazaki D et al (2020) Epidemiological aspects of allergic conjunctivitis. Allergol Int 69(4):487–495. 10.1016/j.alit.2020.06.004 10.1016/j.alit.2020.06.004 32654975

[280] La Rosa M et al (2013) Allergic conjunctivitis: a comprehensive review of the literature. Ital J Pediatr 39:18. 10.1186/1824-7288-39-18 23497516 10.1186/1824-7288-39-18 PMC3640929

[281] Bruschi G, Ghiglioni DG, Cozzi L, Osnaghi S, Viola F, Marchisio P (2023) Vernal keratoconjunctivitis: a systematic review. Clin Rev Allergy Immunol 65(2):277–329. 10.1007/s12016-023-08970-4 37658939 10.1007/s12016-023-08970-4 PMC10567967

[282] Leonardi A, Righetti G, Giovannini G, De Marchi V, Occhiuto M (2023) Diagnostic criteria of chronic conjunctivitis: atopic keratoconjunctivitis and vernal keratoconjunctivitis. Curr Opin Allergy Clin Immunol 23(5):390. 10.1097/ACI.0000000000000915 37284778 10.1097/ACI.0000000000000915

[283] Elieh Ali Komi D, Rambasek T, Bielory L (2018) Clinical implications of mast cell involvement in allergic conjunctivitis. Allergy 73(3):528–539. 10.1111/all.13334 29105783 10.1111/all.13334

[284] Matsuba-Kitamura S et al (2010) Contribution of IL-33 to induction and augmentation of experimental allergic conjunctivitis. Int Immunol 22(6): 479–489. 10.1093/intimm/dxq035 10.1093/intimm/dxq035 20501612

[285] Hosotani Y et al (2023) IL-33-induced keratoconjunctivitis is mediated by group 2 innate lymphoid cells in mice. Allergol Int 72(2):324–331. 10.1016/j.alit.2022.10.003 10.1016/j.alit.2022.10.003 37010996

[286] Fukagawa K, Saito H, Azuma N, Tsubota K, Iikura Y, Oguchi Y (1994) Histamine and tryptase levels in allergic conjunctivitis and vernal keratoconjunctivitis. Cornea 13(4):345–348. 10.1097/00003226-199407000-00010 7924335 10.1097/00003226-199407000-00010

[287] Tesse R et al (2012) New insights into childhood Vernal keratoconjunctivitis-associated factors. Pediatr Allergy Immunol Off Publ Eur Soc Pediatr Allergy Immunol 23(7):682–685. 10.1111/j.1399-3038.2012.01281.x 10.1111/j.1399-3038.2012.01281.x 22963206

[288] Stagi S et al (2014) Increased incidence of thyroid dysfunction and autoimmunity in patients with vernal keratoconjunctivitis. Int J Endocrinol 2014: 804870. 10.1155/2014/804870 10.1155/2014/804870 PMC4130298 25140177

[289] Nebbioso M, Zicari AM, Celani C, Lollobrigida V, Grenga R, Duse M (2015) Pathogenesis of vernal keratoconjunctivitis and associated factors. Semin Ophthalmol 30:5–6. 10.3109/08820538.2013.874483 10.3109/08820538.2013.874483 24571721

[290] Somers EC et al (2016) Antinuclear antibody prevalence in a general pediatric cohort from Mexico City: discordance between immunofluorescence and multiplex assays. Clin Epidemiol 9:1–8. 10.2147/CLEP.S121632 28053555 10.2147/CLEP.S121632 PMC5192054

[291] Yamana Y, Yamana S, Uchio E (2025) Evaluation on the clinical findings and allergological factors of local allergic conjunctivitis and non-local allergic conjunctivitis. Sci Rep 15(1):12566. 10.1038/s41598-025-90273-z 40221483 10.1038/s41598-025-90273-z PMC11998207

[292] Song K, Ye S, Song J, Kang Z (2025) Knowledge attitude and practice of patients with allergic conjunctivitis towards their disease. Sci Rep 15(1):6238. 10.1038/s41598-025-87518-2 39979330 10.1038/s41598-025-87518-2 PMC11842743

[293] Kausar A, Akhtar N, Akbar N (2022) Epidemiological aspects of allergic conjunctivitis. J Ayub Med Coll Abbottabad JAMC 34(1):135–140. 10.55519/JAMC-01-9432 35466641 10.55519/JAMC-01-9432

[294] Leonardi A et al (2013) Vernal Keratoconjunctivitis-like Disease in Adults. Am J Ophthalmol 155(5):796–803. 10.1016/j.ajo.2012.11.018 10.1016/j.ajo.2012.11.018 23352342

[295] Chauhan G et al (2025) Unpacking VKC: How gender and age shape the clinical picture. Indian J Ophthalmol 73(4): 594–598. 10.4103/IJO.IJO_177_25 10.4103/IJO.IJO_177_25 PMC12097409 40146146

[296] Bonini S, Lambiase A, Schiavone M, Centofanti M, Palma LA, Bonini S (1995) Estrogen and progesterone receptors in vernal keratoconjunctivitis. Ophthalmology 102(9):1374–1379. 10.1016/S0161-6420(95)30861-5 9097776 10.1016/s0161-6420(95)30861-5

[297] Sacchetti M, Lambiase A, Moretti C, Mantelli F, Bonini S (2015) Sex hormones in allergic conjunctivitis: altered levels of circulating androgens and estrogens in children and adolescents with vernal keratoconjunctivitis. J Immunol Res 2015(1):945317. 10.1155/2015/945317 25756057 10.1155/2015/945317 PMC4324981

[298] Uchio E, Kimura R, Migita H, Kozawa M, Kadonosono K (2008) Demographic aspects of allergic ocular diseases and evaluation of new criteria for clinical assessment of ocular allergy. Graefes Arch Clin Exp Ophthalmol 246(2):291–296. 10.1007/s00417-007-0697-z 17940788 10.1007/s00417-007-0697-z

